# Carbohydrate metabolism enzymes and phenotypic characterization of diverse lines of the climate‐resilient food, feed, and bioenergy crop *Camelina sativa*

**DOI:** 10.1002/fes3.459

**Published:** 2023-04-14

**Authors:** Peter Stasnik, Johann Vollmann, Dominik K. Großkinsky, Claudia Jonak

**Affiliations:** ^1^ Center for Health and Bioresources, Bioresources Unit AIT Austrian Institute of Technology Konrad‐Lorenz‐Straße 24 3430 Tulln an der Donau Austria; ^2^ Department of Crop Sciences University of Natural Resources and Life Sciences Vienna Konrad‐Lorenz‐Straße 24 3430 Tulln an der Donau Austria

**Keywords:** carbon metabolism, enzyme activities, gold‐of‐pleasure, oilseed crop

## Abstract

Climate change poses tremendous pressure on agriculture. *Camelina sativa* is an ancient, low‐input, high‐quality oilseed crop for food, feed and industrial applications that has retained its natural stress tolerance. Its climate resilience, adaptability to different growth conditions, and the qualities of its seed oil and cake have spurred the interest in camelina. However, due to a period of neglect it has not yet undergone intensive breeding and knowledge about this multi‐purpose crop is still limited. Metabolism is strongly associated with plant growth and development and little information is available on camelina primary carbohydrate metabolism. Here, eight camelina lines from different geographic and climatic regions were characterized for important growth parameters and agricultural traits. Furthermore, the activities of key enzymes of the carbohydrate metabolism were analysed in leaves, seedpods, capsules, and developing seeds. The lines differed in shoot and leaf morphology, plant height, biomass formation as well as in seed yield and seed oil and protein content. Key carbohydrate metabolism enzymes showed specific activity signatures in leaves and reproductive organs during seed development, and different lines exhibited distinct enzyme activity patterns, providing a valuable basis for developing new physiological markers for camelina breeding programs.

## Graphical abstract

In this study, eight camelina lines from different geographic and climatic regions were characterized for growth parameters and relevant agricultural traits. Furthermore, the activities of key enzymes of the carbohydrate metabolism were analysed in leaves, seedpods, capsules, and developing seeds. Principal component analysis (PCA) showed that the lines canbe distinguished based on their carbohydrate enzyme activity profiles. Activities of certain enzymes are associated with growth and yield parameters and thus might contribute as a physiological marker for camelina breeding.

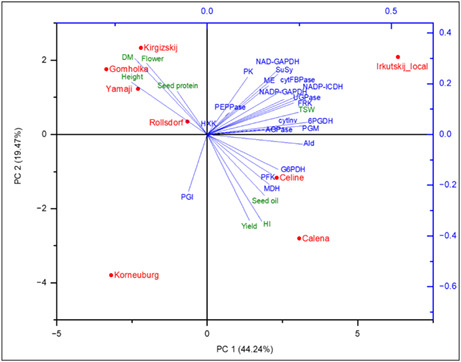

## 1 INTRODUCTION

Climate change increasingly challenges agricultural production systems and thus food and bioenergy security. To meet this challenge, the climate‐resilient, low‐input, versatile Brassicaceae oilseed crop camelina (*Camelina sativa* [L.] Crantz) is a promising alternative species. Camelina, also known as gold‐of‐pleasure, false‐flax, Siberian oilseed, German sesame and linseed dodder is native to Southeast Europe and Southwest Asia and closely related to economically significant crops like *Brassica napus* (rapeseed), *Raphanus sativus* (common radish) and *Armoracia rusticana* (horseradish) as well as the model plant *Arabidopsis thaliana* (thale cress; Faure & Tepfer, 2016; Knörzer, 1978; Vollmann & Eynck, 2015; Zanetti et al., 2017). Camelina is one of the oldest cultivated plants in Europe grown for human consumption and as animal feed starting from 3000 BC. After a period of neglect, camelina is now again increasingly cultivated in Europe, the USA, Canada, Africa, and Asia due to its robustness, its uncommon fatty acid composition, and its great potential across a wide range of applications (Zanetti et al., 2017, 2021).

Camelina seeds contain high levels of polyunsaturated fatty acids and are rich in healthy omega‐3 fatty acids and antioxidants like tocopherol. Its specific qualities make camelina extremely valuable as vegetable oil for human consumption, while the press cake is used as high‐quality animal feed (Berti et al., 2016; Zanetti et al., 2017, 2021). In addition to its food and feed applications, camelina is also used as raw material for various bio‐based applications ranging from cosmetics and soaps to technical lubricants (Berti et al., 2016; Faure & Tepfer, 2016; Zanetti et al., 2017). Also, camelina oil is highly suitable for conversion into renewable biodiesel, which contributes to reducing the use of conventional non‐renewable fuels, highlighting camelina's potential as a multi‐purpose crop (Stamenković et al., 2021).

Camelina can adapt to a wide range of climatic and soil conditions, including marginal lands, and has low water and nutrient input requirements. Camelina has retained its natural stress tolerance and can withstand periods of drought, heat, and low‐temperature stress. It has been shown that the productivity of camelina can compete with that of *Brassica napus* and other mustard species in semi‐dry and cold environmental conditions (Berti et al., 2016, 2017; Fujita et al., 2014; Zanetti et al., 2021). Furthermore, camelina plants are naturally resistant to a number of common biotic stresses, giving it a significant advantage over other *Brassicaceae* crops like rapeseed. For instance, it exhibits resistance to the two diseases that have the greatest global impact on Brassica crop production: blackleg or stem canker disease and Alternaria black spot (Berti et al., 2016; Vollmann & Eynck, 2015; Zanetti et al., 2017).

Camelina is suited for both organic and conventional cropping systems and can be utilized in double‐ and intercropping systems as well as rotational crop in typical agricultural systems due to its short life cycle, its adaptability, and the availability of cultivars tailored to different seasons. Camelina can be integrated into dual crop systems in an economically viable way as a cash cover crop while also providing ecosystem services, especially with the availability of camelina winter ecotypes (Berti et al., 2016; Chen et al., 2015; Faure & Tepfer, 2016; Righini et al., 2016; Zubr, 1997).

The central carbohydrate metabolism is the basis of plant metabolism (Jensen et al., 2007; Martin & Schnarrenberger, 1997; Niyogi et al., 2015; Zeeman, 2015). Photosynthesis converts light energy into chemical energy, which is finally stored in carbohydrate molecules, such as sugars and starch (Gurrieri et al., 2021; Niyogi et al., 2015; Pfister & Zeeman, 2016; Raines, 2003; Zeeman, 2015). The produced carbohydrates can be transported to other organs and fuel further metabolic processes (Koch, 2004). As a consequence, the central carbohydrate metabolism can also be considered as the main pillar of plant growth and production and understanding its processes is an essential basis for crop improvement (Koch, 2004; Niyogi et al., 2015; Zeeman, 2015). The activities of enzymes involved in central carbohydrate metabolism arise through the integration of a variety of regulatory mechanisms, including transcriptional, post‐transcriptional, and post‐translational processes and have been shown to be associated with growth performance, crop yield, and quality. As determinants of a plant's physiological state, enzymatic activities of plant metabolism emerge for characterization and differentiation of plant genotypes and their metabolic and agronomic properties (Cañas et al., 2017; Fernandez et al., 2016; Jammer et al., 2015; Pandey et al., 2021; Steinhauser et al., 2010; Stitt & Gibon, 2014).

Camelina has the potential to be a productive crop for future agriculture, supporting adaptation of agricultural cropping systems to increasingly difficult conditions and providing important fatty acids and proteins for food, feed, and bio‐based industries. However, as a result of the long period of neglect, our basic knowledge of camelina is still limited. Despite its highly favourable qualities for cultivation in different environmental conditions, including challenging ones, and as a raw material for bio‐based industries, as well as its excellent nutritional profile for humans and animals, camelina and its products still occupy a niche in agriculture and industry. Thus, to promote the deployment of camelina as multi‐purpose crop in sustainable agriculture and to further exploit it as a future climate smart crop, it is important to increase our knowledge about agronomic and physiological properties of camelina. In this study, we investigated whether specific enzymatic signatures are associated with different camelina lines and their agronomic traits. For this, agricultural parameters of eight different camelina lines were analysed, and the enzymatic activities of key carbohydrate metabolism enzymes were assessed in different plant organs. The study combined morphological and physiological phenotyping (Großkinsky et al., 2015), which can serve as a basis for developing this promising climate‐resilient crop to better meet future food and energy security challenges.

## 2 MATERIALS AND METHODS

### 2.1 Plant material

Eight camelina (*Camelina sativa* [L.] Crantz) lines of diverse geographical/climatic origin across Eurasia and different breeding background were compared (Table 1). The selection was based on Vollmann et al. (2005), which categorized a set of 130 camelina accessions into four groups with different seed characteristics. At least one line from each of these four groups was selected for the experiments.

**TABLE 1:** Investigated *Camelina sativa* lines, their geographic origins, leaf margin and branch morphology (Hotton et al., 2020; Vollmann et al., 2005).

Line	Origin	Breeding history	Leaf margin	Branch pattern
Calena	Austria	Cultivar	Serrate	X/Y‐type
Celine	France	Cultivar	Spiny	W‐type
Kirgizskij	Kyrgyzstan	Landrace	Spiny	X/Y‐type
Irkutskij local	Russia	Landrace	Spiny	X/Y‐type
Gomholka	Hungary	Landrace	Serrate	W‐type
Rollsdorf	Austria	Landrace	Smooth	X/Y‐type
Korneuburg	Austria	Landrace	Serrate	W‐type
Yamaji	China	Landrace	Smooth	W‐type

### 2.2 Seed sterilization and pot preparation

The seeds were surface sterilized with chlorine (10 g L^−1^ in 90% ethanol) for 8 min, rinsed three times with 96% ethanol and air dried overnight. The sterilized seeds were sown about 1 cm deep in 1.452 L plastic pots with dimensions 11 × 11 × 12 cm (Göttinger). The pots were filled with soil‐peat substrate (Einheitserde special Profisubstrat SP ED63; PATZER ERDEN GmbH). Soil was treated with nematodes (Biohelp Garten & Bienen) once before sowing to prevent fly pests. Four seeds per pot were sown and one seedling was retained after thinning at the stage BBCH 101 (2 leaf stage).

### 2.3 Growth conditions and experimental setup

Camelina plants were grown in the greenhouse with 16 h supplementary illumination by MASTER SON‐T PIA Plus 400W sodium bulbs (Philips) for about 3 months till maturity. In each of the two independent experiments, a total of eight plants per line were cultivated, i.e., eight replications in a randomized complete block (RCB) experimental design. Plants were watered twice per week. Information about leaf morphology, leaf shape morphology and branching phenotype were collected and characterized according to Hotton et al. (2020). The leaf morphology was classified into the “lanceolate” leaf shape (narrow oval shape tapering to a point at each end), the “subulate” leaf shape (slender and tapering to a point) and the “linear” leaf shape (long and very narrow like a blade of grass). To avoid senescing leaves, the characterization was done mainly in the vegetative phase at around BBCH 120 where the clearest individual phenotypes were observed, and once the plants had bolted and reached their final height. In the vegetative phase, all lines showed leaves of the “lanceolate” leaf shape. Towards the end of their life cycle all plants developed in the upper part smaller, thinner, and darker leaves of the “subulate” shape. At that time, older leaves were already highly senescent and could not be characterized any more.

The leaf margin morphology was classified into the three groups “spiny” (stiff, sharp points such as thistles), “serrated” (saw‐toothed with asymmetrical teeth pointing forward) or “smooth” (even margin without points).

The branching phenotype was classified into four groups, which are the “W type” (branched only at top), the “X type” (branched down length of the main stem), the “Y type” (most of the secondary branches originating from the base) and the “Z type” (no main stem observed and secondary branches emerging everywhere). The characterization of the branching phenotype was done once the plants had bolted and reached their final height.

For the physiological phenotyping and enzymatic measurements, leaves and seedpods were harvested and pooled from three plants per line. For the leaf harvest, only still‐green leaves from the main stem of the plant between leaf number 18 and 22 were chosen. For the seedpod harvest, two different seedpod stages/sizes were harvested: small/younger (green) seedpods in an early developmental phase with less than 6 mm in length and less than 4 mm in width and big/older (green) seedpods in a late developmental phase with more than 6 mm in length and more than 4 mm in width. In addition, some of the older seedpods were also divided into capsules and developing seeds, which were analysed separately.

Harvesting was performed before maturity during the fruit development and seed‐filling phase of the still‐green seedpods around BBCH 705, according to the established BBCH scale for *Camelina sativa* (Martinelli & Galasso, 2011). Samples were snap frozen in liquid nitrogen and stored at −80°C till use. The remaining plants were grown to maturity. At full maturity stage, the plants were cut, harvested, and separated into seeds and straw. Two times two hundred seeds per plant were counted with a Contador seed counter (Pfeuffer GmbH) and weighted to determine the thousand seed weight (TSW). The remaining biomass was dried at 65°C until weight remained constant to determine the weight of the dry matter. The data of the total seed yield and the dry matter weight were used to calculate the Harvest Index (HI).

### 2.4 Near‐infrared reflectance (NIR) spectroscopy

Oil and protein contents of the sieved seeds were quantified using Fourier transform near‐infrared reflectance spectroscopy (Bruker MATRIX™‐I FT‐NIR spectrometer; Bruker) as described in Vollmann et al. (2005). About 5–10 g of dry camelina seeds were used for the non‐destructive measurement, and all measurements were carried out twice. The NIR device was earlier calibrated with camelina reference samples (*n* = 222 samples for oil; *n* = 120 samples for protein) from eight different environments. Calibration models were based on partial least square regressions (PLSR) with validation *R* ^2^ values of *R* ^2^ = 91.5 for oil and *R* ^2^ = 95.7 for protein.

### 2.5 Extraction of enzymes

The extraction of enzymes was based on Gibon et al. (2004) with some modifications. The material was collected from about 55‐day‐old plants and ground to a fine powder in liquid nitrogen and instantly used for extraction and analysis. For the extraction, 75 mg of the powder with 0.1% polyvinylpolypyrrolidone (PVPP) was extracted in 500 μL of extraction buffer by vortexing for 15 min at 4°C and centrifugation for 30 min at 4°C at 16,200× *g*. The extraction buffer contained 50 mM Hepes/KOH, pH 7.5, 10% (v/v) glycerol, 0.1% (v/v) Triton X‐100, 10 mM MgCl_2_, 1 mM EDTA, 1 mM dithiothreitol (DTT), 1 mM benzamidine, 1 mM ε‐aminocaproic acid, 1 mM phenylmethylsulfonylfluoride (PMSF), and 10 mM leupeptin. Leupeptin and PMSF were added freshly before extraction. The extracts were purified using PD MiniTrap™ G‐25 Sephadex columns (Cytiva) equilibrated by adding 6 mL of 50 mM Hepes/KOH, pH 7.5 buffer and 1 mL of enzyme extraction buffer. Subaliquots were diluted further to generate appropriate dilutions for the different enzymes. The amount of extract and the dilution was chosen according to the individual enzyme and the type of material to maintain the reaction in the linear range.

### 2.6 Kinetic enzyme activity assays

The protocols were based on Gibon et al. (2004) and Jammer et al. (2015) with adaptions and modifications. All kinetic enzyme activity assays were performed in a 96‐well microtiter plate format for semi‐high‐throughput application. For routine measurement, 10 μL of the different protein extracts in a total reaction volume of 200 μL (with 190 μL of reaction mixture containing buffer components, substrates, auxiliary substances, and auxiliary enzymes) were incubated in UV‐transmissive flat bottom 96‐well plates (UV‐Star^R^ microplate; Greiner Bio One) in a plate reader (CLARIOstar, BMG LABTECH GmbH) at 30°C for 20–30 min and absorbance at 260 nm for NAD(P) and 340 nm for NAD(P)H was monitored throughout the entire period of incubation. For control reactions, substrate was omitted. For Blanks, extraction buffer was used instead of extract. The change in absorbance during the linear phase of the kinetic reaction was used as the basis for the calculation of specific enzyme activity in µmol min^‐1^g^−1^ FW or in µmol min^‐1^mg^−1^ protein.

ADP‐glucose pyrophosphorylase (AGPase; EC 2.7.7.27) was assayed by incubating the extracts in a reaction mixture containing 5 mM MgCl_2_, 0.4 mM EDTA, 2 mM 3‐PGA, 1 mM NADP, 1.5 mM PPi, 2 mM ADP‐glucose, 0.02 mM G‐1,6‐BP, 2.5 UmL^−1^ PGM and 5 UmL^−1^ G6PDH in 100 mM Tricine/KOH buffer at pH 8.0. For control reactions, ADP‐glucose was omitted.

Aldolase (Ald; EC 4.1.2.13) was assayed by incubating the extracts in a reaction mixture containing 5 mM MgCl_2_, 1 mM EDTA, 1 mM Fru‐1,6‐BP, 0.2 mM NADH, 2.5 UmL^−1^ TPI, 5 UmL^−1^ G3PDH in 100 mM Tricine/KOH buffer at pH 8.0. For control reactions, Fru‐1,6‐BP was omitted.

Cytosolic fructose‐1,6‐bisphosphatase (cytFBPase; EC 3.1.3.11) was assayed by incubating the extracts in a reaction mixture containing 2 mM MgCl_2_, 0.4 mM NADP, 0.2 mM Fru‐1,6‐BP, 2 UmL^−1^ PGI and 2 UmL^−1^ G6PDH in 50 mM HEPES/KOH buffer at pH 7.0. For control reactions, fructose‐1,6‐bisphosphate was omitted.

Fructokinase (FRK; EC 2.7.1.4) was assayed by incubating the extracts in a reaction mixture containing 5 mM MgCl_2_, 3 mM fructose, 1 mM NADP, 1.5 mM ATP, 2 UmL^−1^ PGI and 2 UmL^−1^ G6PDH in 100 mM Tricine/KOH buffer at pH 8.0. For control reactions, ATP was omitted.

Glucose‐6‐phosphate dehydrogenase (G6PDH; EC 1.1.1.49) was assayed by incubating the extracts in a reaction mixture containing 10 mM MgCl_2_, 0.5 mM NADP and 3 mM G6P in 100 mM Tricine/KOH buffer at pH 8.0. For control reactions, G6P was omitted.

NAD‐dependent glyceraldehyde 3‐phosphate dehydrogenase (NAD‐GAPDH; EC 1.2.1.12) was assayed by incubating the extracts in a reaction mixture containing 30 mM MgCl_2_, 20 mM KCl, 2 mM EDTA, 5 mM DTT, 6 mM 3PGA, 2 mM ATP, 0.3 mM NADH and 5 UmL^−1^ PGK in 100 mM Tricine/KOH buffer at pH 8.0. For control reactions, NADH was omitted.

NADP‐dependent glyceraldehyde 3‐phosphate dehydrogenase (NADP‐GAPDH; EC 1.2.1.12) was assayed by incubating the extracts in a reaction mixture containing 30 mM MgCl_2_, 20 mM KCl, 2 mM EDTA, 5 mM DTT, 6 mM 3PGA, 2 mM ATP, 0.3 mM NADH and 5 UmL^−1^ PGK in 100 mM Tricine/KOH buffer at pH 8.0. For control reactions, NADH was omitted.

Hexokinase (HXK; EC 2.7.1.1) (Glucokinase activity) was assayed by incubating the extracts in a reaction mixture containing 5 mM MgCl_2_, 5 mM glucose, 1 mM NADP, 1.5 mM ATP and 2 UmL^−1^ G6PDH in 100 mM Tricine/KOH buffer at pH 8.0. For control reactions, ATP was omitted.

NADP‐dependent isocitrate dehydrogenase (NADP‐ICDH; EC 1.1.1.41) was assayed by incubating the extracts in a reaction mixture containing 4 mM MgCl_2_, 1 mM NADP and 2 mM isocitrate in 100 mM Tricine/KOH buffer at pH 8.0. For control reactions, isocitrate was omitted.

Cytosolic invertase (cytInv; EC 3.2.1.26) was assayed by incubating the extracts in a reaction mixture containing 5 mM MgCl_2_, 1 mM EDTA, 1 mM ATP, 0.5 mM NADP, 100 mM sucrose, 2.5 UmL^−1^ HXK, 2 UmL^−1^ PGI and 2.5 UmL^−1^ G6PDH in 50 mM HEPES/KOH buffer at pH 7.0. For control reactions, sucrose was omitted.

NADP‐dependent malic enzyme (NADP‐ME; EC 1.1.1.38) was assayed by incubating the extracts in a reaction mixture containing 5 mM MgCl_2_, 1 mM NADP and 3 mM malate in 50 mM HEPES/KOH buffer at pH 7.5. For control reactions, malate was omitted.

NADP‐dependent malate dehydrogenase (NADP‐MDH; EC 1.1.1.37) was assayed by incubating the extracts in a reaction mixture containing 5 mM MgCl_2_, 1 mM oxalacetate and 0.3 mM NADPH in 50 mM HEPES/KOH buffer at pH 7.5. For control reactions, oxalacetate was omitted.

PEP phosphatase (PEPPase; EC 3.1.3.60) was assayed by incubating the extracts in a reaction mixture containing 10 mM MgCl_2_, 50 mM KCl, 1 mM DTT, 2 mM PEP, 0.3 mM NADH and 2.5 UmL^−1^ lactate dehydrogenase in 50 mM HEPES/KOH buffer at pH 7.0. For control reactions, PEP was omitted.

Phosphofructokinase (PFK; EC 2.7.1.11) was assayed by incubating the extracts in a reaction mixture containing 5 mM MgCl_2_, 1 mM EDTA, 1 mM ATP, 2 mM F6P, 0.2 mM NADH, 2.5 UmL^−1^ aldolase, 2.5 UmL^−1^ TPI, 5 UmL^−1^ G3PDH in 100 mM Tricine/KOH buffer at pH 8.0. For control reactions, F6P was omitted.

6‐phosphogluconase dehydrogenase (6PGDH; EC 1.1.1.44) was assayed by incubating the extracts in a reaction mixture containing 10 mM MgCl_2_, 0.5 mM NADP and 3 mM 6PG in 100 mM Tricine/KOH buffer at pH 8.0. For control reactions, 6PG was omitted.

Phosphoglucose isomerase (PGI; EC 5.3.1.9) was assayed by incubating the extracts in a reaction mixture containing 5 mM MgCl_2_, 4 mM DTT, 0.5 mM NADP, 2 mM F6P and 2.5 UmL^−1^ G6PDH in 100 mM Tricine/KOH buffer at pH 8.0. For control reactions, F6P was omitted.

Phosphoglucose mutase (PGM; EC 5.4.2.2) was assayed by incubating the extracts in a reaction mixture containing 5 mM MgCl_2_, 4 mM DTT, 0.5 mM NADP, 0.02 mM Glu‐1,6‐BP, 2 mM G1P and 2.5 UmL^−1^ G6PDH in 100 mM Tricine/KOH buffer at pH 8.0. For control reactions, G1P was omitted.

Pyruvate kinase (PK; EC 2.7.1.40) was assayed by incubating the extracts in a reaction mixture containing 10 mM MgCl_2_, 50 mM KCl, 1 mM DTT, 1 mM ADP, 2 mM PEP, 0.3 mM NADH and 2.5 UmL^−1^ lactate dehydrogenase in 50 mM HEPES/KOH buffer at pH 7.0. For control reactions, PEP was omitted. The assay was corrected for PEP phosphatase background activity.

Sucrose synthase (SuSy; EC 2.4.1.13) was assayed by incubating the extracts in a reaction mixture containing 5 mM MgCl_2_, 1 mM EDTA, 1 mM UDP, 1 mM ATP, 0.5 mM NADP, 100 mM sucrose, 2.5 UmL^−1^ HXK, 2 UmL^−1^ PGI and 2.5 UmL^−1^ G6PDH in 50 mM HEPES/KOH buffer at pH 7.0. For control reactions, sucrose was omitted. The assay was corrected for cytosolic invertase background activity.

UDP‐glucose pyrophosphorylase (UGPase; EC 2.7.7.9) was assayed by incubating the extracts in a reaction mixture containing 5 mM MgCl_2_, 0.4 mM EDTA, 2 mM 3‐PGA, 1 mM NADP, 2.5 mM PPi, 2 mM UDP‐glucose, 0.02 mM G‐1,6‐BP, 2.5 UmL^−1^ PGM and 5 UmL^−1^ G6PDH in 100 mM Tricine/KOH buffer at pH 8.0. For control reactions, UDP‐glucose was omitted.

### 2.7 Protein quantification

Protein contents of the extracts were quantified based on the principle of Bradford with the ready‐made ROTI™Quant solution (Carl Roth GmbH + Co. KG) in a 96‐well microplate format according to the manufacturer's manual.

### 2.8 Statistical analysis

Results of the morphological and agronomic traits including yield, dry matter, height and thousand seed weight (TSW) were mainly evaluated in Microsoft Excel. The correlation analysis was also performed in Microsoft Excel. The primary results of the kinetic measurements were evaluated and pre‐processed in MARS Data Analysis Software from BMG Labtech. Normal distribution and homogeneity of variances of datasets have been checked using diagnostic plots in R Studio. The subsequent ANOVA and Tukey's Honest Significant Difference (HSD) Post‐Hoc tests (alpha = 0.05) were also done in R Studio. Activity data from 20 different enzymes and the combination of enzyme activity and agronomic data (28 traits in total) were further utilized to classify camelina lines in multivariate statistical analyses through principal components (PCA). Enzyme data from different developmental stages and different organs, normalized to FW or protein content, were standardized (*z*‐transformation) and analysed separately. Vector loadings of traits, scores of camelina lines, and biplots were calculated and visualized using OriginPro Version 2023 software (OriginLab Corp.).

## 3 RESULTS

### 3.1 Line‐specific differences of camelina growth and biomass formation

Eight *Camelina sativa* cultivars and landraces from different geographic regions were selected from a study by Vollmann et al. (2005), which categorized a set of 130 camelina accessions into four groups according to their seed characteristics. At least one line from each of the four defined groups was characterized. Two lines (Calena and Celine) are cultivars that have undergone a breeding programme, while the other lines represent landraces with little or no selection history (Table 1).

**FIGURE 1:**
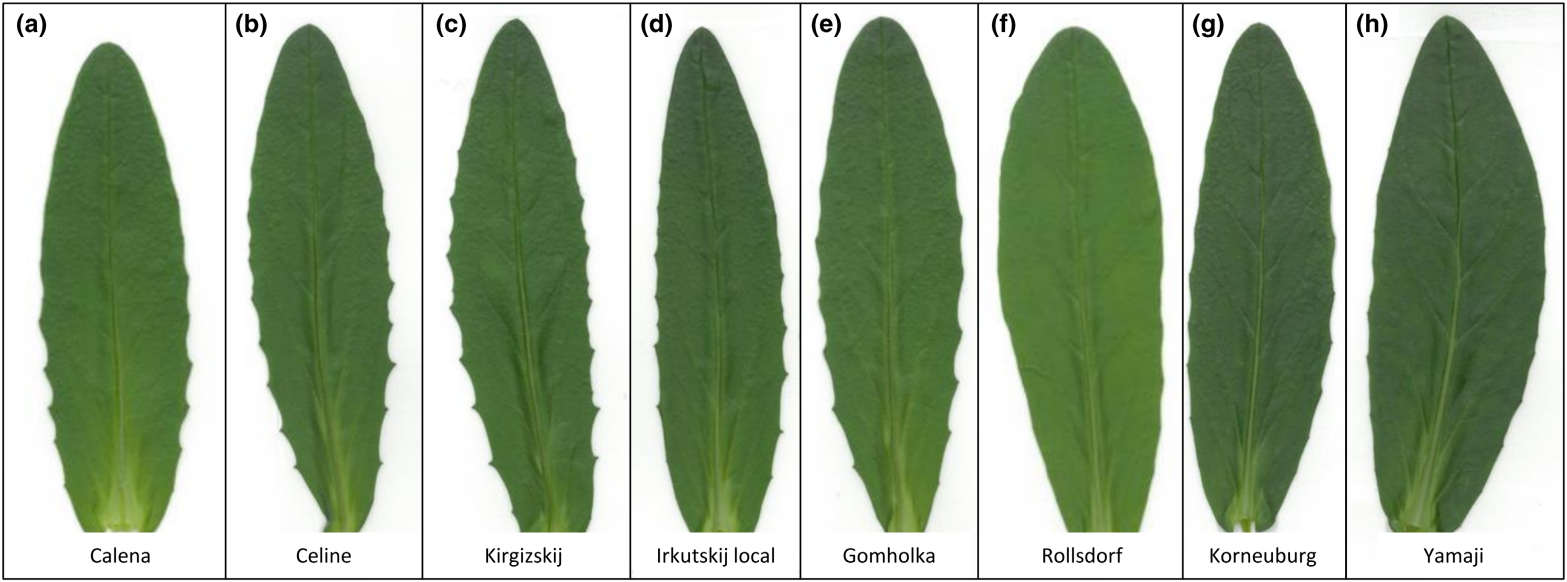
Leaf shape and margins of different *Camelina sativa* lines. A representative picture of a leaf between leaf number 11–13 in the vegetative phase (BBCH 116–118) before the onset of flowering of (a) Calena, (b) Celine, (c) Kirgizskij, (d) Irkutskij local, (e) Gomholka, (f) Rollsdorf, (g) Korneuburg and (h) Yamaji is shown.

The eight selected camelina lines displayed differed morphological phenotypes when grown in the greenhouse under long‐day conditions. Different lines showed line‐specific leaf shapes and margins (Figure 1; Table 1) and were classified according to Hotton et al. (2020). In the vegetative phase, all lines had “lanceolate” leaves. Towards the end of their life cycle, all plants developed smaller, thinner, and darker leaves in the upper part with a “subulate” shape. By this time, older leaves were already highly senescent and could not be characterized any more. While the older leaves in the lower part of the plant had overall “smooth” leaf margins, the younger and middle‐aged leaves towards the top of the plant showed line‐specific differences in their leaf margins. Of the tested lines, Celine, Kirgizskij and Irkutskij local showed a “spiny” leaf margin, Calena, Gomholka and Korneuburg showed a “serrated” leaf margin and Rollsdorf and Yamaji showed a “smooth” leaf margin without any points.

Shoot branching is determined by endogenous and environmental factors and plays a central role in the development of the above‐ground plant structure which affects crop productivity. In our experimental setup, different camelina lines showed different branching phenotypes and were classified according to Hotton et al. (2020). The majority of plants of the lines Celine, Gomholka, Korneuburg and Yamaji exhibited a W‐type phenotype, while plants of the line Calena, Kirgizskij, Irkutskij local and Rollsdorf showed a X‐type or a Y‐type phenotype (Table 1).

**FIGURE 2:**
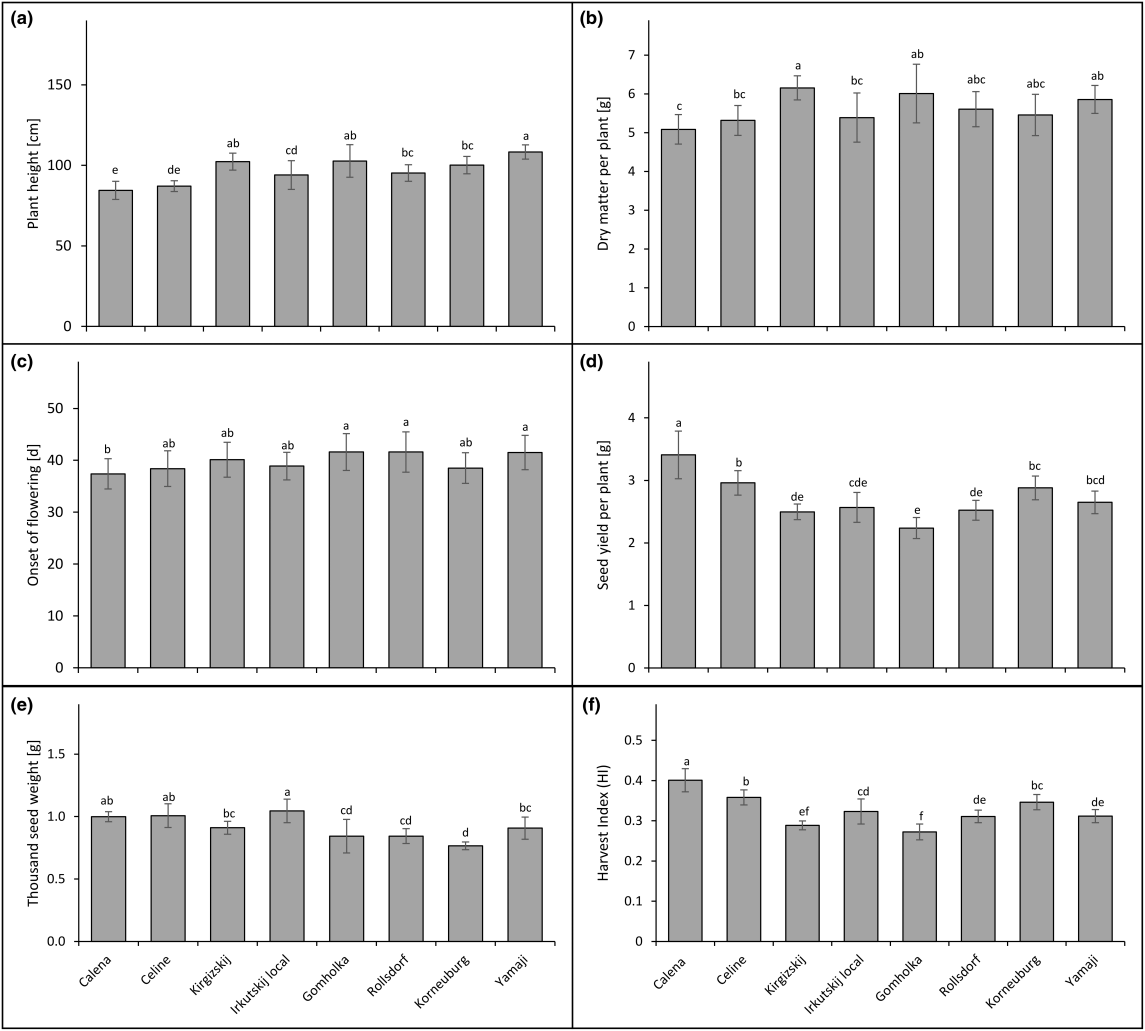
Line‐specific differences in agronomic and developmental parameters. Means, SDs, and ANOVA with Tukey's Honest Significant Difference (HSD) Test of eight *Camelina sativa* cultivars and landraces with 16 biological replicates each for (a) plant height, (b) dry matter per plant, (c) onset of flowering, (d) yield per plant, (e) thousand seed weight and f) Harvest Index. Significant differences (alpha = 0.05) between lines are indicated by different letters.

The tested camelina lines significantly varied in the final plant height (Figure 2a), dry matter biomass formation (Figure 2b) and in the time to flowering (Figure 2c). The two cultivars, Calena and Celine, were significantly shorter than the landraces. Plants of the lines Yamaji, Gomholka and Kirgizskij grew tallest. Dry matter biomass formation ranged from 6.21 to 5.09 g per plant (Figure 2b). Kirgizskij, Gomholka, Yamaji and Rollsdorf formed the highest dry matter biomass, ranging from 6.21 to 5.61 g per plant. Korneuburg and Irkutskij local showed an intermediate dry matter biomass formation of 5.46 and 5.39 g per plant. Celine and Calena formed the lowest dry matter biomass (5.32 and 5.09 g per plant).

Under the given conditions, Calena started flowering earliest (on average 37 days after sowing) followed by Celine (on average 38 days), Korneuburg and Irkutskij local (on average 39 days). Kirgizskij started flowering after about 40 days. Yamaji, Gomholka and Rollsdorf were the latest to start flowering on average with 42 days after sowing (Figure 2c).

### 3.2 Seeds of the camelina lines differ in quantitative and quality parameters

To estimate the agronomic performance of the tested camelina lines, we determined the seed yield per plant (Figure 2d), the thousand seed weight (TSW; Figure 2e) and the harvest index (HI; Figure 2f). Calena had the overall highest seed yield with 3.41 g per plant. Celine, Korneuburg and Yamaji showed the second highest seed yield per plant (ranging from 2.96 to 2.65 g) which was significantly lower than that of Calena. Seed yield per plant for Irkutskij local was 2.57 g, for Rollsdorf 2.52 g and for Kirgizskij 2.49 g. Gomholka showed the lowest seed yield (2.2 g per plant) compared to the other lines tested.

HI is an important agronomic parameter of crop yield. It is defined as the seed weight divided by the total weight of above‐ground biomass. Of the eight camelina lines tested under the given conditions, Calena had the significantly highest HI of 0.40. Celine and Korneuburg had the second highest HI of 0.36 and 0.35, respectively, followed by Irkutskij local (0.32) and Yamaji (0.31). Rollsdorf, Kirgizskij and finally Gomholka showed the lowest HI (ranging from 0.30 to 0.27) among all tested lines. As a measure of seed size, the TSW (thousand seed weight) was determined (Figure 2e). In our experiment, Irkutskij local, Calena and Celine had the highest TSW followed by Yamaji, Kirgizskij, Rollsdorf, and Gomholka. Korneuburg showed the lowest TSW.

To get insights into the qualitative composition of the seeds of the different camelina lines, the seed oil and protein content was estimated by near‐infrared reflectance spectroscopy (NIRS; Table 2). The analysis of the oil content in the seeds showed that Celine, Korneuburg and Irkutskij local accumulated the highest amount of oil, followed by Rollsdorf and Calena. Gomholka had significantly less oil compared to Celine, Korneuburg and Irkutskij local. Kirgizskij and Yamaji showed the lowest oil content of all lines tested. As for the protein content in the seeds, Gomholka showed the highest protein level. Kirgizskij, Irkutskij local, Korneuburg, Yamaji, Celine and Calena had intermediate protein content in the seeds. Rollsdorf had the lowest seed protein content and differed significantly from Gomholka. Camelina can be used as both oil and protein feedstock. Under the given conditions, the observed oil:protein ratio ranged from 1.19 to 1.00 in the different lines tested. Rollsdorf and Celine exhibited the highest oil:protein ratio (1.19 and 1.18, respectively) while Kirgizskij showed the lowest oil:protein ratio (1.00).

**TABLE 2:** Oil content and protein content [mg g^−1^] of dried seeds for eight *Camelina sativa* lines with 16 biological replicates.

	Oil content			Protein content			Oil:Protein ratio
	Average	SD	Group	Average	SD	Group	
Calena	340.0	10.8	ab	295.0	15.0	ab	1.15
Celine	357.1	36.4	a	302.2	26.0	ab	1.18
Kirgizskij	311.7	28.6	c	310.9	22.6	ab	1.00
Irkutskij local	349.9	14.6	a	309.8	17.5	ab	1.13
Gomholka	324.7	24.2	bc	317.9	25.5	a	1.02
Rollsdorf	345.6	17.6	ab	290.6	9.4	b	1.19
Korneuburg	350.8	17.2	a	308.6	30.2	ab	1.14
Yamaji	309.8	26.2	c	304.2	25.1	ab	1.02

### 3.3 Carbohydrate metabolic enzymes exhibit organ‐specific activity patterns

**FIGURE 3:**
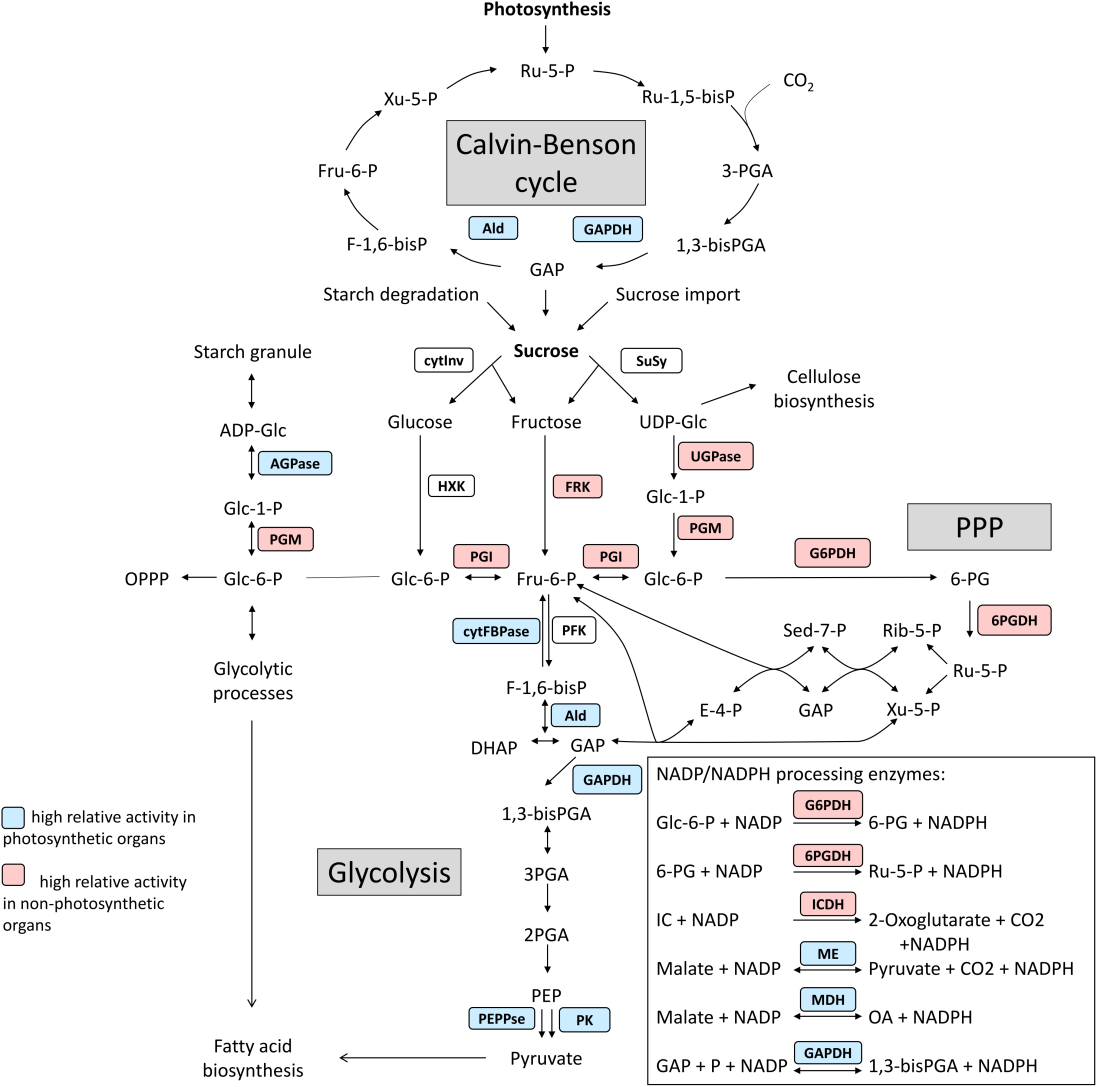
Schematic overview of key enzymes in primary carbohydrate metabolism in plants. Differences in relative activity between photosynthetic and non‐photosynthetic organs are indicated by colour. AGPase, ADP‐glucose pyrophosphorylase; cytInv, cytosolic invertase; SuSy, sucrose synthase; Ald, aldolase; NAD‐GAPDH, NAD‐dependent glyceraldehyde 3‐phosphate dehydrogenase; NADP‐GAPDH, NADP‐dependent glyceraldehyde 3‐phosphate dehydrogenase; cytFBPase, cytosolic fructose 1,6‐bisphosphatase; NADP‐ICDH, NADP‐dependent isocitrate dehydrogenase; FRK, fructokinase; PGI, phosphoglucose isomerase; PGM, phosphoglucose mutase; UGPase, UDP‐glucose pyrophosphorylase; HXK, hexokinase; 6PGDH, 6‐phosphogluconate dehydrogenase; G6PDH, glucose 6‐phosphate dehydrogenase; PFK, phosphofructokinase; NADP‐ME, NADP‐dependent malic enzyme; NADP‐MDH, NADP‐dependent malate dehydrogenase; PEPPase, phosphoenolpyruvate phosphatase; PK, pyruvate kinase. Colour code: blue background of enzyme slot, high relative activity of enzyme in photosynthetic organs; red background of enzyme slot, high relative activity of enzyme in non‐photosynthetic organs.

**FIGURE 4:**
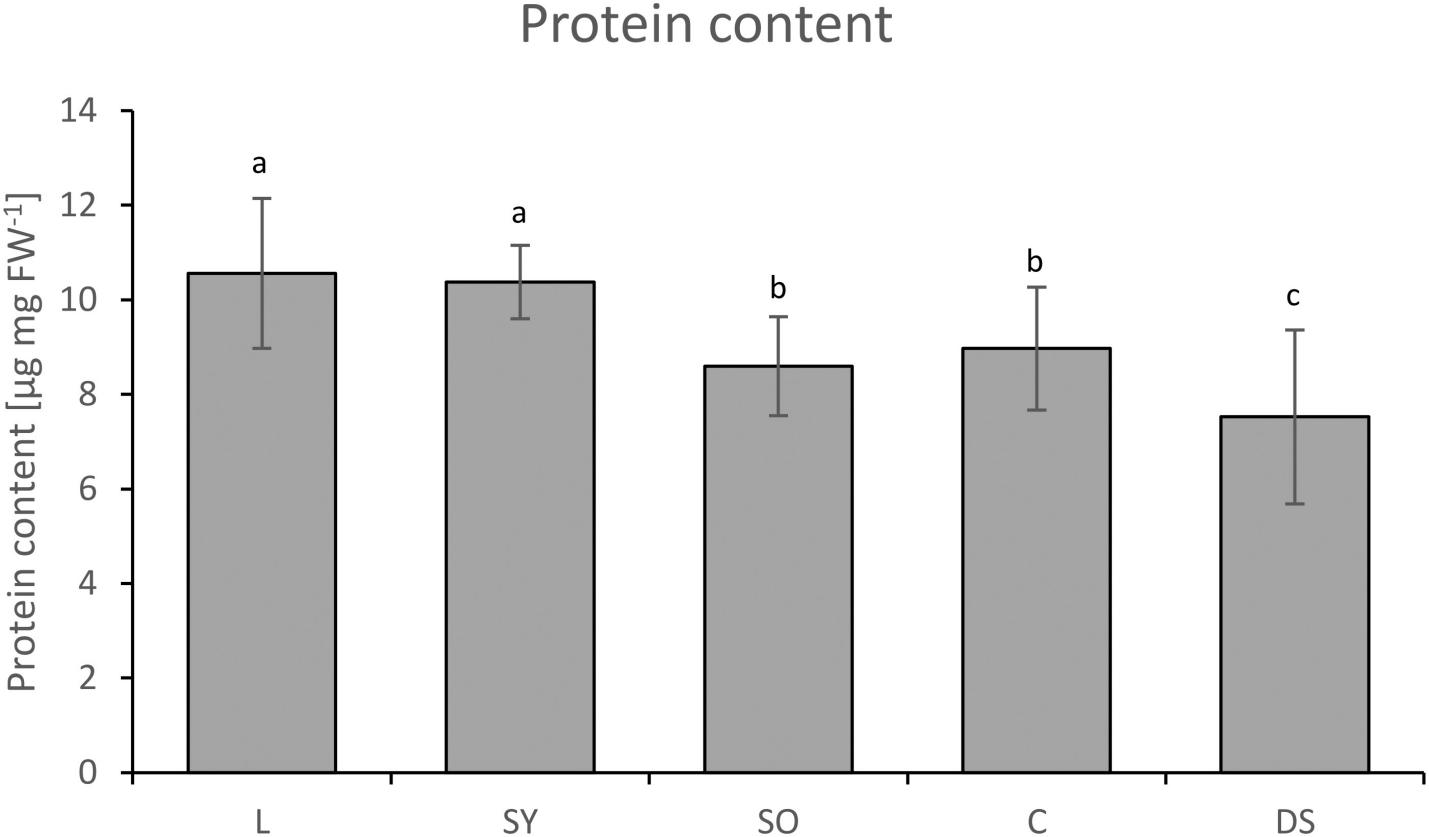
Organ‐specific differences in total protein extracts. C, capsules; DS, developing seeds; FW, fresh weight; L, leaves; SO, seedpods old; SY, seedpods young.

To gain insight into metabolic processes during the ripening and seed‐filling phase, we analysed the activity of 20 different key enzymes from the central carbohydrate metabolism in leaves, young and old seedpods as well as from developing seeds and capsules (i.e., deseeded seedpods) of plants at approximately BBCH 705. The young seedpods were at the stage when oil and protein production are substantial. The old seedpods were at a more mature stage, when the seedpods had reached their final size but were still green. As the capsules are photosynthetically active, the old seedpods were divided into capsules and developing seeds. Selected enzymes included key regulatory enzymes from glycolysis, glyconeogenesis, the Calvin‐Benson cycle, the oxidative pentose phosphate cycle as well as sucrose and starch processing enzymes (Figure 3). The measured enzyme activities of the different organs of the different camelina lines were normalized to both fresh weight and to protein content to account for different protein levels in the sampled organs. On average across all lines, leaves and young seedpods showed the highest protein content while old seedpods had a lower protein content (Figure 4). Capsules generally contained more protein than the developing seeds, which showed the lowest protein content of all analysed plant parts. A similar pattern was observed in all tested lines with the exception of Korneuburg which showed a high protein content in developing seeds (Figure S1).

**FIGURE 5:**
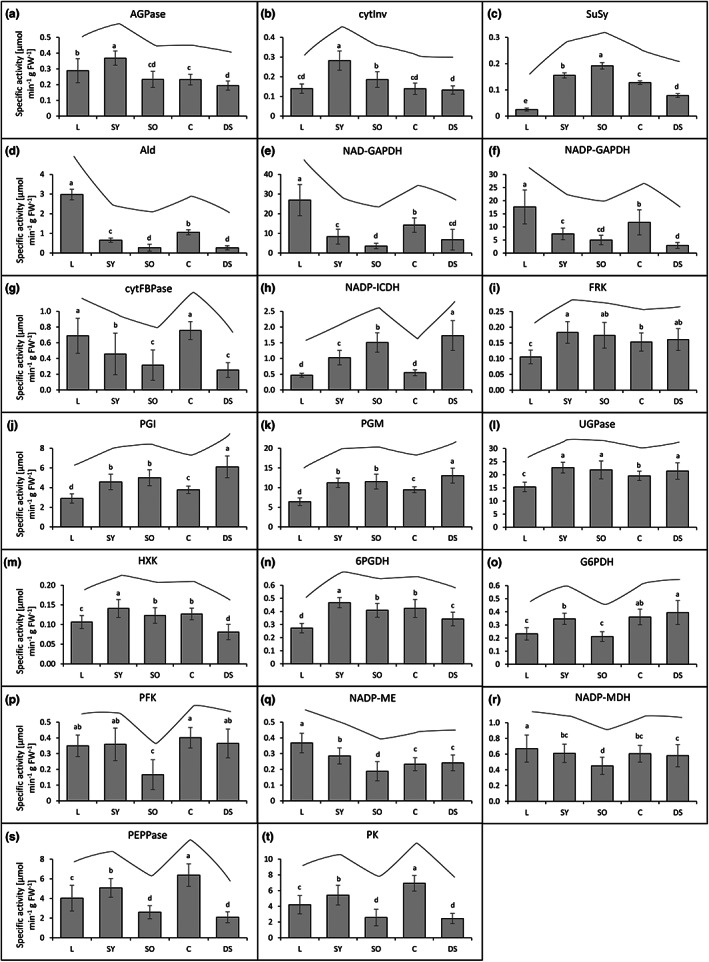
Specific activities of central carbohydrate metabolic enzymes averaged over eight *Camelina sativa* lines and normalized to the extracted fresh weight (FW). Organ‐specific enzyme activity patterns are visualized in the respective diagrams above the bars. Means, SDs, and ANOVA with Tukey's Honest Significant Difference (HSD) Test of enzymatic activities of leaves (L), young seedpods (SY), old seedpods (SO), capsules (C), and developing seeds (DS) over eight different *Camelina sativa* lines. (a) ADP‐glucose pyrophosphorylase (AGPase; EC 2.7.7.27), (b) cytosolic invertase (cytInv; EC 3.2.1.26), (c) sucrose synthase (SuSy; EC 2.4.1.13), (d) aldolase (Ald; EC 4.1.2.13), (e) NAD‐dependent glyceraldehyde 3‐phosphate dehydrogenase (NAD‐GAPDH; EC 1.2.1.12), (f) NADP‐dependent glyceraldehyde 3‐phosphate dehydrogenase (NADP‐GAPDH; EC 1.2.1.12), (g) cytosolic fructose 1,6‐bisphosphatase (cytFBPase; EC 3.1.3.11), (h) NADP‐dependent isocitrate dehydrogenase (NADP‐ICDH; EC 1.1.1.41), (i) fructokinase (FRK; EC 2.7.1.4), (j) phosphoglucose isomerase (PGI; EC 5.3.1.9), (k) phosphoglucose mutase (PGM; EC 5.4.2.2), (l) UDP‐glucose pyrophosphorylase (UGPase; EC 2.7.7.9), (m) hexokinase (HXK; EC 2.7.1.1), (n) 6‐phosphogluconate dehydrogenase (6PGDH; EC 1.1.1.44), (o) glucose 6‐phosphate dehydrogenase (G6PDH; EC 1.1.1.49), (p) phosphofructokinase (PFK; EC 2.7.1.11), (q) NADP‐dependent malic enzyme (NADP‐ME; EC 1.1.1.38), (r) NADP‐dependent malate dehydrogenase (NADP‐MDH; EC 1.1.1.37), (s) phosphoenolpyruvate phosphatase (PEPPase; EC 3.1.3.60), (t) pyruvate kinase (PK; EC 2.7.1.40).

**FIGURE 6:**
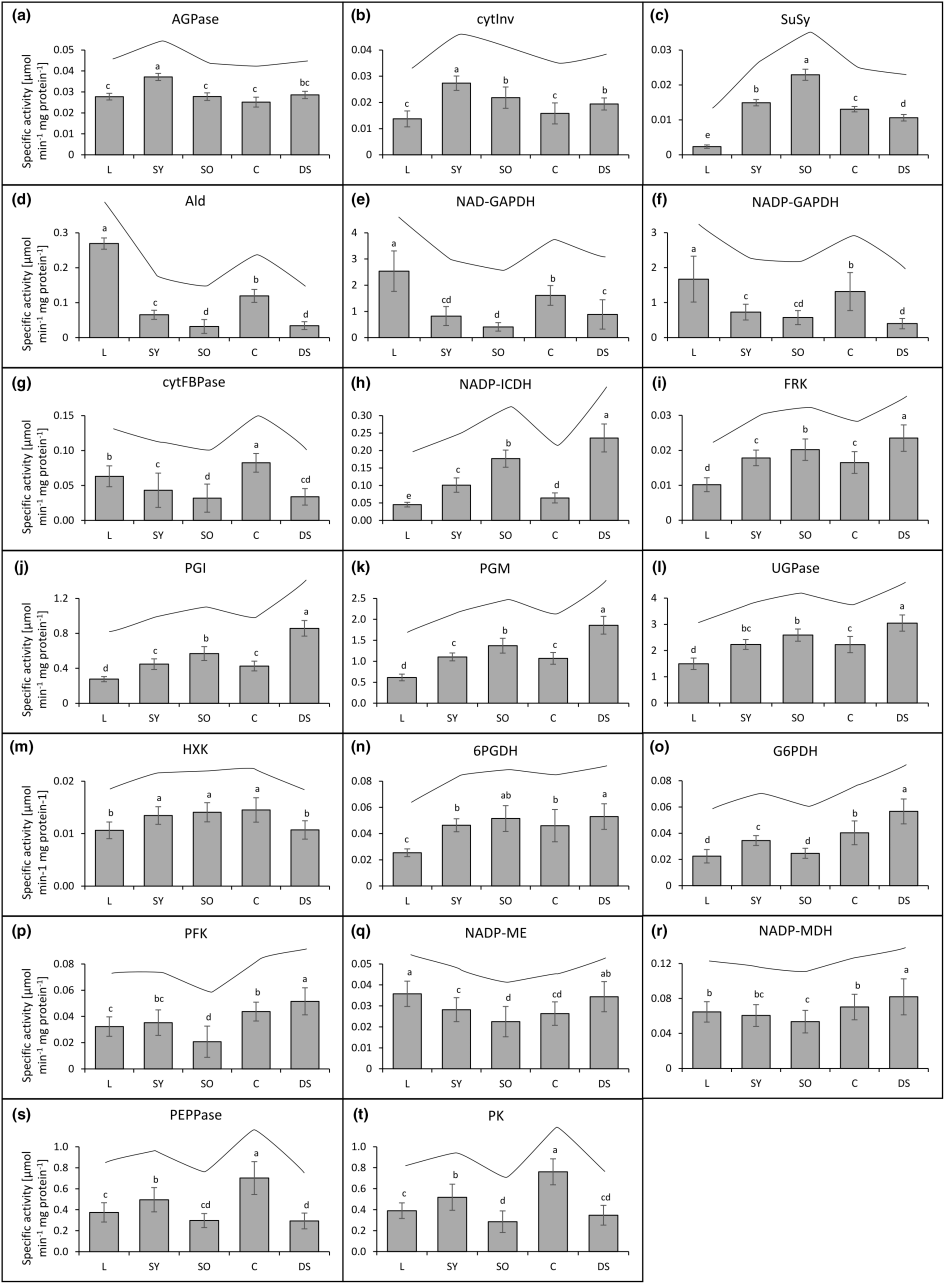
Specific activities of central carbohydrate metabolic enzymes averaged over eight *Camelina sativa* lines normalized to the total protein content. Organ‐specific enzyme activity patterns are visualized in the respective diagrams above the bars. Means, SDs, and ANOVA with Tukey's Honest Significant Difference (HSD) Test of enzymatic activities of leaves (L), young seedpods (SY), old seedpods (SO), capsules (C), and developing seeds (DS) over eight different *Camelina sativa* lines. (a) ADP‐glucose pyrophosphorylase (AGPase; EC 2.7.7.27), (b) cytosolic invertase (cytInv; EC 3.2.1.26), (c) sucrose synthase (SuSy; EC 2.4.1.13), (d) aldolase (Ald; EC 4.1.2.13), (e) NAD‐dependent glyceraldehyde 3‐phosphate dehydrogenase (NAD‐GAPDH; EC 1.2.1.12), (f) NADP‐dependent glyceraldehyde 3‐phosphate dehydrogenase (NADP‐GAPDH; EC 1.2.1.12), (g) cytosolic fructose 1,6‐bisphosphatase (cytFBPase; EC 3.1.3.11), (h) NADP‐dependent isocitrate dehydrogenase (NADP‐ICDH; EC 1.1.1.41), (i) fructokinase (FRK; EC 2.7.1.4), (j) phosphoglucose isomerase (PGI; EC 5.3.1.9), (k) phosphoglucose mutase (PGM; EC 5.4.2.2), (l) UDP‐glucose pyrophosphorylase (UGPase; EC 2.7.7.9), (m) hexokinase (HXK; EC 2.7.1.1), (n) 6‐phosphogluconate dehydrogenase (6PGDH; EC 1.1.1.44), (o) glucose 6‐phosphate dehydrogenase (G6PDH; EC 1.1.1.49), (p) phosphofructokinase (PFK; EC 2.7.1.11), (q) NADP‐dependent malic enzyme (NADP‐ME; EC 1.1.1.38), (r) NADP‐dependent malate dehydrogenase (NADP‐MDH; EC 1.1.1.37), (s) phosphoenolpyruvate phosphatase (PEPPase; EC 3.1.3.60), (t) pyruvate kinase (PK; EC 2.7.1.40).

First, to get a general overview, the activities of the various central carbohydrate metabolism enzymes in leaves and reproductive organs were averaged across all lines. The activity patterns of the analysed enzymes of the different organs were similar when normalized to either fresh weight (Figure 5) or protein content (Figure 6) indicating robustness. Several enzymes showed similar activity patterns in the different organs analysed and could be grouped into different activity pattern groups.

ADP‐glucose pyrophosphorylase (AGPase) and cytosolic invertase (cytInv) activities were highest in young seedpods compared to the other organs tested (Figures 5a,b and 6a,b). Sucrose synthase (SuSy), another sucrolytic enzyme besides invertases, showed a similar pattern with a high activity in young seedpods and an even higher activity in old seedpods and a very low activity in leaves (Figures 5c and 6c).

Aldolase (Ald), NAD‐dependent glyceraldehyde 3‐phosphate dehydrogenase (NAD‐GAPDH) and NADP‐dependent glyceraldehyde 3‐phosphate dehydrogenase (NADP‐GAPDH) showed the highest activity in leaves compared to all other analysed organs (Figures 5d–f and 6d–f). The activities of these enzymes were higher in young than in old seedpods and higher in capsules than in seeds. Cytosolic fructose 1,6‐bisphosphatase (cytFBPase) showed a similar pattern but with comparable activities in leaves and capsules (Figures 5g and 6g).

NADP‐dependent isocitrate dehydrogenase (NADP‐ICDH) showed an activity pattern inverse to NADP‐GAPDH. Its activity was lowest in leaves and capsules, increasingly high from the younger to the older seedpods, and highest in developing seeds (Figures 5h and 6h).

Activities of fructokinase (FRK), phosphoglucose isomerase (PGI), phosphoglucose mutase (PGM), and UDP‐glucose pyrophosphorylase (UGPase) were significantly higher in reproductive organs than in leaves (Figures 5i–l and 6i–l) with a higher activity in developing seeds than in capsules, which was particularly pronounced for PGI and PGM.

Hexokinase (HXK) activity was higher in seedpods and capsules as compared to leaves and developing seeds. 6‐phosphogluconate dehydrogenase (6PGDH) showed a similar activity pattern (Figures 5m,n and 6m,n).

Glucose 6‐phosphate dehydrogenase (G6PDH) activity was highest in developing seeds and its activity was higher in young than in old seedpods and leaves (Figures 5o and 6o). Likewise, phosphofructokinase (PFK), NADP‐dependent malic enzyme (NADP‐ME) and NADP‐dependent malate dehydrogenase (NADP‐MDH) had a low activity in old seedpods compared to the other organs (Figures 5p–r and 6p–r). NADP‐ME and NADP‐MDH showed high activity in leaves.

Phosphoenolpyruvate phosphatase (PEPPase) and pyruvate kinase (PK) activities were highest in capsules while their activities in the developing seeds were very low compared to the other analysed organs (Figures 5s,t and 6s,t). Their activities were higher in the young than in old seedpods.

Generally, there was a pattern that most of the sucrose degrading enzymes and enzymes of the initial steps of glycolysis showed a low relative activity in photosynthetic leaves and capsules, in comparison to seeds. In contrast, enzymes of the later steps of glycolysis, glyconeogenesis and the Calvin‐Benson cycle showed a high relative activity in non‐photosynthetic organs compared to photosynthetic organs (Figure 3).

The organ‐specific activities of the analysed enzymes followed the same general trends in all tested camelina lines, with only few line‐specific differences (Figures S2 and S3). However, many of these individual differences were dependent on the normalization, due to the differences in the relative protein content between the lines. Examples of significant line‐specific differences independent of the type of normalization include the HXK activity in seedpods and developing seeds which showed considerably lower activity in Calena, Celine, Kirgizskij and Irkutskij local compared to Rollsdorf, Korneuburg and Yamaji. Furthermore, Korneuburg and Yamaji showed outstandingly high FRK activity in many organs compared to the other lines, while the G6PDH activity was high in Irkutskij local, Rollsdorf and Yamaji. PGI and PGM activities were the lowest in the seedpods and developing seeds in Calena, Celine and Irkutskij local.

### 3.4 Enzymatic activities allow differentiation between camelina lines

To get an overview and further insight into the differences of the enzyme activities between the different camelina lines and their association with agronomic traits, principal component analyses (PCA) were performed. PCA of the enzyme activities of the different organs and developmental stages showed that the lines can be distinguished based on their carbohydrate enzyme activity profiles (Figure S4). Calena and Celine were closely linked, especially in early developmental stages (leaves and young siliques) and were also grouped in the same quadrant in PCA based on old seedpods and developing seeds. Rollsdorf and Korneuburg appeared to be most different to these two commercial lines in most organs and developmental stages.

**FIGURE 7:**
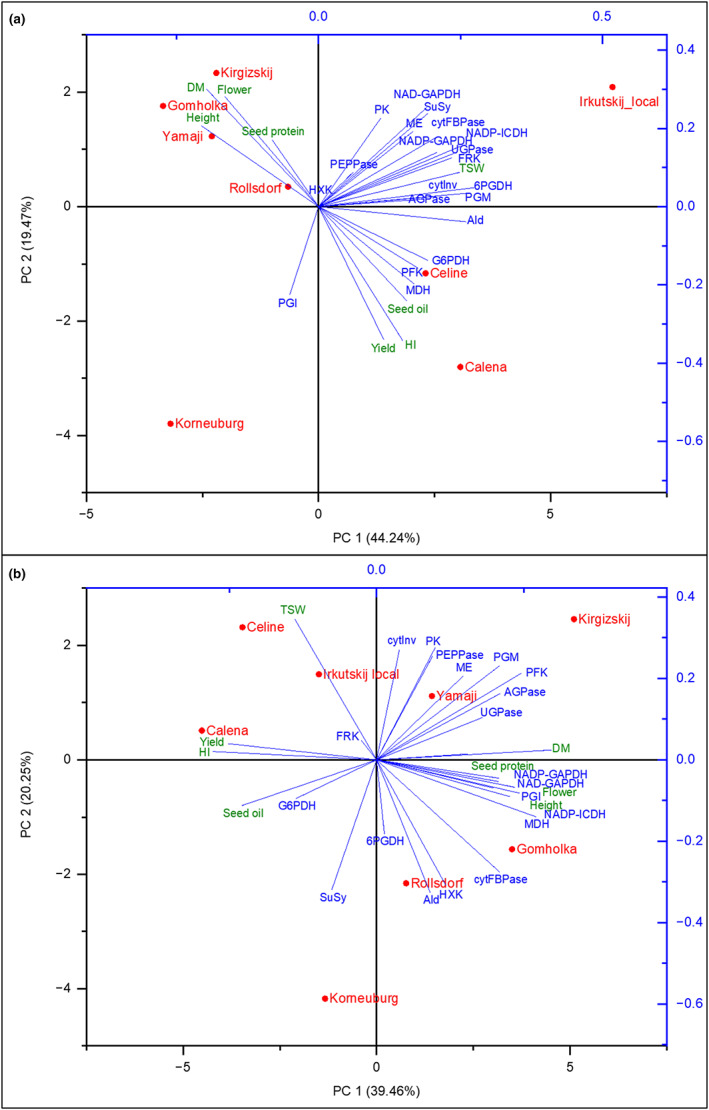
Principal component analysis of different *Camelina sativa* lines based on agronomic and enzyme activity data normalized to fresh weight. PCA biplots were performed with enzyme activity data from leaves (a) and developing seeds (b). The percentages of total variance represented by principal component 1 (PC1) and principal component 2 (PC2) are shown in parentheses. Loading scores of *Camelina sativa* lines are depicted in red; vector loadings of individual enzyme activities are shown in blue, vector loadings of the agronomic parameters are shown in green. 6PGDH, 6‐phosphogluconate dehydrogenase; AGPase, ADP‐glucose pyrophosphorylase; Ald, aldolase; cytFBPase, cytosolic fructose 1,6‐bisphosphatase; cytInv, cytosolic invertase; DM, dry matter biomass; Flower, onset of flowering; FRK, fructokinase; G6PDH, glucose 6‐phosphate dehydrogenase; Height, Plant height; HI, Harvest index; HXK, hexokinase; MDH, NADP‐dependent malate dehydrogenase; ME, NADP‐dependent malic enzyme; NAD‐GAPDH, NAD‐dependent glyceraldehyde 3‐phosphate dehydrogenase; NADP‐GAPDH, NADP‐dependent glyceraldehyde 3‐phosphate dehydrogenase; NADP‐ICDH, NADP‐dependent isocitrate dehydrogenase; PEPPase, phosphoenolpyruvate phosphatase; PFK, phosphofructokinase; PGI, phosphoglucose isomerase; PGM, phosphoglucose mutase; PK, pyruvate kinase; Seed oil, seed oil content; Seed protein, seed protein content; SuSy, sucrose synthase; TSW, thousand seed weight; UGPase, UDP‐glucose pyrophosphorylase.

PCA based on agronomic data and enzyme activities in leaves and developing seeds, respectively, revealed a negative correlation between yield and seed protein content, and between seed oil and seed protein content (Figure 7 and Figure S5). Seed protein content clustered with flowering time, dry matter, and plant height. Calena and Celine correlated with yield, HI, and seed oil content. These two commercial lines were further away from the enzyme activities, while the landraces generally were more closely associated with the enzyme activities, suggesting that the landraces might have retained a higher metabolic plasticity as compared to Calena and Celine. Remarkably, G6PDH activity was associated with seed oil content not only in developing seeds but also in leaves. To a lower extent, also NADP‐MDH and PFK in leaves were associated with yield, seed oil content and HI. Figures [Link], [Link] provide detailed correlations of enzyme activities with individual agronomic parameters in different organs and developmental stages.

## 4 DISCUSSION

Current agricultural systems are challenged by more frequent and severe adverse climatic conditions while also being under pressure to boost production and product quality. In many regions of the world, climate change has already resulted in large yield losses and is expected to become worse in the future (Hübner & Kantar, 2021). Due to its innate tolerance to various types of stress, including heat and drought, the underutilized oilseed crop camelina could contribute to addressing these issues (Berti et al., 2016; Chen et al., 2015; Righini et al., 2016; Zubr, 1997). Camelina has the potential to be a productive climate‐smart crop for future agriculture, supporting sustainable cropping systems and supplying critical fatty acids for food, feed, and energy security. However, because it has long been neglected, both considerable research to improve basic knowledge of camelina and breeding efforts are required to achieve these goals. In this study, comparative analysis of growth behaviour and productivity parameters together with activity signatures of selected key enzymes of carbohydrate metabolism of different camelina lines provided evidence that enzymatic activities are associated with agronomic parameters. Furthermore, different camelina lines could be distinguished based on their specific enzyme activity patterns.

The eight camelina lines, which included cultivars and landraces from different geographic regions, showed differences in morphologic parameters, such as plant height, branching and leaf margin, as well as in agronomic parameters, such as seed yield, thousand seed weight (TSW) and seed oil and seed protein content. The cultivars differed from the landraces by having a lower plant height and dry matter biomass and a higher yield. There was also some variation among the lines in seed oil and protein content, TSW and onset of flowering but there was no clear division by previous breeding activities. These results deriving from greenhouse‐grown camelina lines are consistent with a previous study in a fully controlled chamber (Stasnik et al., 2022). The eight lines tested showed similar performance with Calena, Celine and Korneuburg having the highest yield while Kirgizskij, Rollsdorf and Gomholka showed a low yield but high dry matter biomass. Seed yield and dry matter biomass formation were generally higher in this compared to the previous study, which could be explained by the differences in pot volume and thus resource availability (Poorter et al., 2012). It is worth noting that plant height was comparable in both studies, suggesting that the differences in available resources did not significantly impact final plant height. The onset of flowering was accelerated compared to Stasnik et al. (2022), which could be a result of the increased substrate and nutrient availability (Kumar & Chaudhary, 2018; McConnaughay & Bazzaz, 1991) and/or the different light and temperature conditions in the greenhouse (Goto et al., 1991; Thomas, 2006).

The lines analysed in this work were selected from a field study that grouped diverse camelina lines into four different groups according to their seed characteristics. The seed quality characters of the different greenhouse‐grown camelina lines were comparable to those of the field trials in terms of their ranking and also reflected the grouping as described in Vollmann et al. (2005). In line with the field experiments, Irkutskij local, Celine and Calena showed a high TSW, while Gomholka, Rollsdorf, Korneuburg and Yamaji had a low TSW. Kirgizskij performed worse compared to the field study. Similar to the TSW, the content of oil and protein in the seeds were comparable to what was found in Vollmann et al. (2005). In both studies, seed oil content was highest in Calena, Celine, Irkutskij local, Korneuburg and Rollsdorf and lowest in Kirgizskij, Gomholka and Yamaji. The opposite pattern was found for the seed protein content, which was also consistent with what was found in Vollmann et al. (2005), except for Yamaji that showed lower protein levels. While the ranking of the greenhouse‐grown lines by the seed characters was comparable to that of the field trials, the differences between the lines in the greenhouse were less pronounced than in the field study, which might be due to a significant environmental influence on seed quality.

Metabolic pathways involving specific activities of metabolic enzymes are of central importance for the maintenance and regulation of developmental processes and thus for plant growth and yield formation. Hence, we measured the activity of 20 different key enzymes from the central carbohydrate metabolism in this study and related them to the observed phenotypes. In general, the analysed enzymes showed similar activity patterns in the different lines. In seeds, starch and oil are used as storage compounds for the next generation and are mainly synthesized from sucrose, imported from maternal tissue (Albacete et al., 2014; Baud & Graham, 2006; Koley et al., 2022; Stitt & Sonnewald, 1995). Sucrose can be metabolized in two different pathways, either by sucrose synthase (SuSy) or invertases (Inv) (Stitt & Sonnewald, 1995). Both enzymes, SuSy and cytInv, showed much lower activity in leaves than in reproductive organs and were negatively correlated in developing seeds in line with the latter being active sucrose sinks (Baud & Graham, 2006; Greiner et al., 1998; Koley et al., 2022). A similar activity pattern of SuSy and Inv has been reported in the *Brassicaceae* crop canola (King et al., 1997).

The enzymes UDP‐glucose pyrophosphorylase (UGPase), hexokinase (HXK) and fructokinase (FRK) convert the hexoses produced into hexose phosphates and fill the hexose phosphate pool, while phosphoglucose isomerase (PGI) and phosphoglucose mutase (PGM) keep the hexose phosphate pool in equilibrium. Interestingly, these enzymes showed a similar activity pattern. FRK, PGI and PGM activities show the best agreement among these five enzymes in organ‐specific relative activity patterns, especially when related to the total protein content, with low relative activity in the leaves, increasing activity in the seedpods over time and high activity in the seeds. PGI and PGM activities were particularly high in the developing seeds compared to the other organs. The high activities of these enzymes, that are involved in many different processes, including synthesis and breakdown of sucrose and starch, glycolysis, and general carbohydrate biosynthesis (Thomas et al., 1992) might be correlated with high biosynthetic rates in developing reproductive organs (Degani et al., 1992; Mazarei et al., 2003; Thomas et al., 1992).

Overall, most enzymes involved in sucrose degradation and the initial steps of glycolysis, including SuSy, FRK, UGPase, PGI and PGM, showed a low relative activity in photosynthetic organs (leaves and capsules) but a high relative activity in non‐photosynthetic organs (seeds). Likewise, the NADPH‐producing enzymes glucose 6‐phosphate dehydrogenase (G6PDH), 6‐phosphogluconate dehydrogenase (6PGDH) from the oxidative pentose phosphate pathway and the NADP‐dependent isocitrate dehydrogenase (NADP‐ICDH) also followed this trend, which might be correlated with the increased demand of energy and reduction equivalents in developing seeds.

On the other hand, enzymes of the later steps of glycolysis and glyconeogenesis, such as cytosolic fructose 1,6‐bisphosphatase (cytFBPase), aldolase (Ald), pyruvate kinase (PK) and PEP phosphatase (PEPPase), had a high activity in photosynthetic organs (leaves and capsules) compared to non‐photosynthetic organs (seeds). Likewise, the activities of the NADPH processing enzymes NAD‐dependent glyceraldehyde 3‐phosphate dehydrogenase (NAD‐GAPDH), NADP‐dependent glyceraldehyde 3‐phosphate dehydrogenase (NADP‐GAPDH), NADP‐dependent malic enzyme (NADP‐ME), and NADP‐dependent malate dehydrogenase (NADP‐MDH), catalysing reversible reactions and/or consuming NADPH, were high in leaves and capsules compared to seeds. It is worth noting that many of these enzymes are involved in several processes. For example, isoforms of Ald and GAPDHs have not only a role in glycolysis, but also in the Calvin‐Benson cycle, explaining the high activity of these enzymes in photosynthetic active organs, such as leaves or capsules (Figure 3).

PK and PEPPase showed a specific enzyme activity pattern with a low activity in old seedpods and developing seeds compared to other organs studied. Since their product pyruvate is required for acetyl‐CoA and fatty acid synthesis in seeds, this might seem unexpected at first sight (Andre et al., 2007). However, it is consistent with a decrease in the rate of fatty acid synthesis as the seeds mature (Simcox et al., 1979). Furthermore, in *Arabidopsis thaliana* the gene expression of pyruvate dehydrogenase, the enzyme that converts pyruvate into acetyl‐CoA, is high in flowers and young siliques but drops drastically in older siliques (Ke et al., 2000). Generally, most of the measured enzymes showed a higher activity in young than in old seedpods. However, to dissect the dynamics of enzyme activities in relation to seed development processes in more detail, analyses with higher spatio‐temporal resolution and determination of the precise developmental stages are necessary.

When analysing the activities of the different carbohydrate metabolism enzymes in the seeds of the compared lines, it is noticeable that lines which have a particular yield characteristic have similar relative activities for certain enzymes. For example, Calena, Celine and Korneuburg showed the highest seed yield per plant and compared to other lines, and a low activity in ADP‐glucose pyrophosphorylase (AGPase), PGI, PGM, phosphofructokinase (PFK), UGPase and NADP‐GAPDH in developing seeds. On the other hand, Kirgizskij, Gomholka (and Rollsdorf) showed a low seed yield per plant and exhibited overall high relative activities for these enzymes in the developing seeds.

Further, Calena, Celine, and Irkutskij local had the highest TSW and a high seed oil content and showed the overall lowest activities for HXK, PGI, PGM, NADP‐ICDH, NADP‐MDH, and NADP‐ME in developing seeds. Interestingly these lines were classified into the same group in the field study described by Vollmann et al. (2005) which might indicate physiological similarities. Gomholka, Kirgizskij and Yamaji had a low seed oil content and high PGI, PGM and PFK activities in the seeds. In line with our data, seeds of a low oil content sunflower variety showed higher activities of the carbohydrate and glycolytic enzymes HXK and PGI compared to a standard oil content variety (Troncoso‐Ponce et al., 2010).

Principal component analysis (PCA) of the enzymatic activities provided an overview of differentiation between the compared camelina lines in basic physiological processes as revealed by activities of 20 enzymes representing different key metabolic pathways. Interestingly, effects of selection were visible on the levels of enzyme activity signatures consistent with the selection history. In PCA of agronomic parameters and enzyme activities, Calena and Celine were further away from most enzymes, while landraces generally were more closely associated with the enzymes, suggesting that landraces have retained a higher metabolic plasticity as compared to the commercial lines Calena and Celine.

The similarity of Calena and Celine, which have been selected under central/west European conditions for yield and seed oil quality traits, was also evident in PCA of enzyme activities. Similarity is most evident in leaves and early stages of seedpod development suggesting physiological differentiation by differential enzyme activities at later stages such as the seed‐filling stage or between capsule and developing seed tissues.

Agronomic characters are not representing one particular sampling stage but instead reveal accumulative processes across all stages. Thus, combining agronomic characters and enzyme activities at different stages can identify particular processes relevant for yield or seed oil accumulation at a given developmental stage. In PCA including enzymatic and agronomic data, yield, harvest index (HI) and seed oil content clustered together and were negatively correlated with dry matter biomass formation, height, onset of flowering and seed protein content. These correlations are in line with that of other crops including *Brassica napus* (Chang et al., 2022; Marjanović‐Jeromela et al., 2011; Stolte et al., 2022) and the negative correlation between seed oil content and seed protein content reported for camelina (Vollmann et al., 2005). It is worth noting that the correlations observed were not only evident in developing seeds but also in leaves, underpinning that physiological process throughout development influence agronomic characters.

The correlation between G6PDH and seed oil content, yield, HI, and TSW in both PCA and correlation analyses is of particular interest highlighting major physiological processes involved in yield and oil accumulation. A crucial role of G6PDH, the key enzyme of the oxidative pentose phosphate pathway, for fatty acid synthesis and seed filling has previously been discussed for different plants such as *Brassica napus* and *Arabidopsis thaliana* (Andre et al., 2007; Focks & Benning, 1998; Hutchings et al., 2005). While the correlation was strongest in seedpods and developing seeds, a remarkable correlation of G6PDH with seed oil content, yield and HI was also found in leaves, indicating that G6PDH activity might contribute as a physiological marker for camelina breeding.

Taken together, the combined analysis of camelina developmental and agronomic parameters with enzymes of the carbohydrate metabolism suggests that combinations of certain enzyme activities can be associated with yield parameters. Further studies with more lines and a more refined time‐course are required to establish and expand specific enzyme signatures as promising future markers supporting camelina breeding.

## 5 CONCLUSION

Among eight camelina lines tested, there was variation in shoot and leaf morphology, flowering time, plant height and dry matter as well as seed yield, oil content and thousand seed weight. Evaluation of key enzymes of the carbohydrate metabolism revealed specific activity signatures in leaves and reproductive organs during the seed development phase. Furthermore, analysis of the activities of the different enzymes in the seeds showed that lines with particular yield characteristics had similar relative activities for combinations of specific enzymes. Overall, the data presented provide a valuable basis for developing new physiological markers for camelina breeding programs to improve its productivity for supporting the availability of climate‐resilient food, feed, and biofuel.

## AUTHOR CONTRIBUTIONS

Genetic material was provided by JV. PS and DKG designed and performed the experiments. PS, DKG, JV, and CJ analysed the data and prepared the manuscript. All authors read and approved the manuscript.

## CONFLICT OF INTEREST STATEMENT

The authors have stated explicitly that there are no conflicts of interest in connection with this article.

## Supporting information


Figure S1

Figure S2

Figure S3

Figure S4

Figure S5

Figure S6

Figure S7

Figure S8

Figure S9

Figure S10

Figure S11

## Data Availability

The material used in this study is available for non‐commercial research purposes upon reasonable request.
