# Supplementary figures and images for: Carbohydrate metabolism enzymes and phenotypic characterization of diverse lines of the climate‐resilient food, feed, and bioenergy crop Camelina sativa
Source: Food Energy Secur. 2023 Apr 14;12(3):e459. doi: 10.1002/fes3.459 (PMC10909413; doi:10.1002/fes3.459)

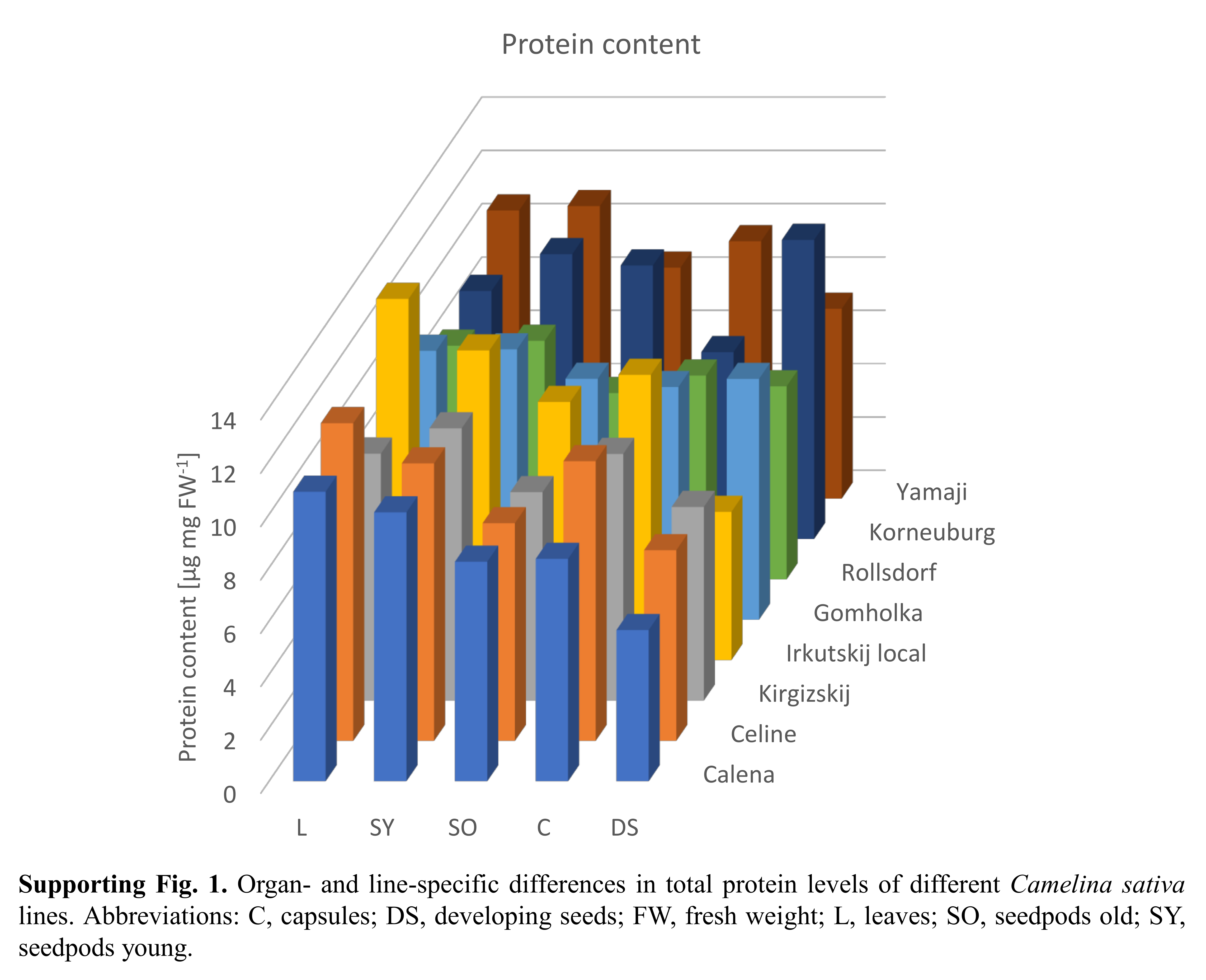

Supplement: Supplementary file 1 — Figure S1 [file FES3-12-e459-s001.tif]

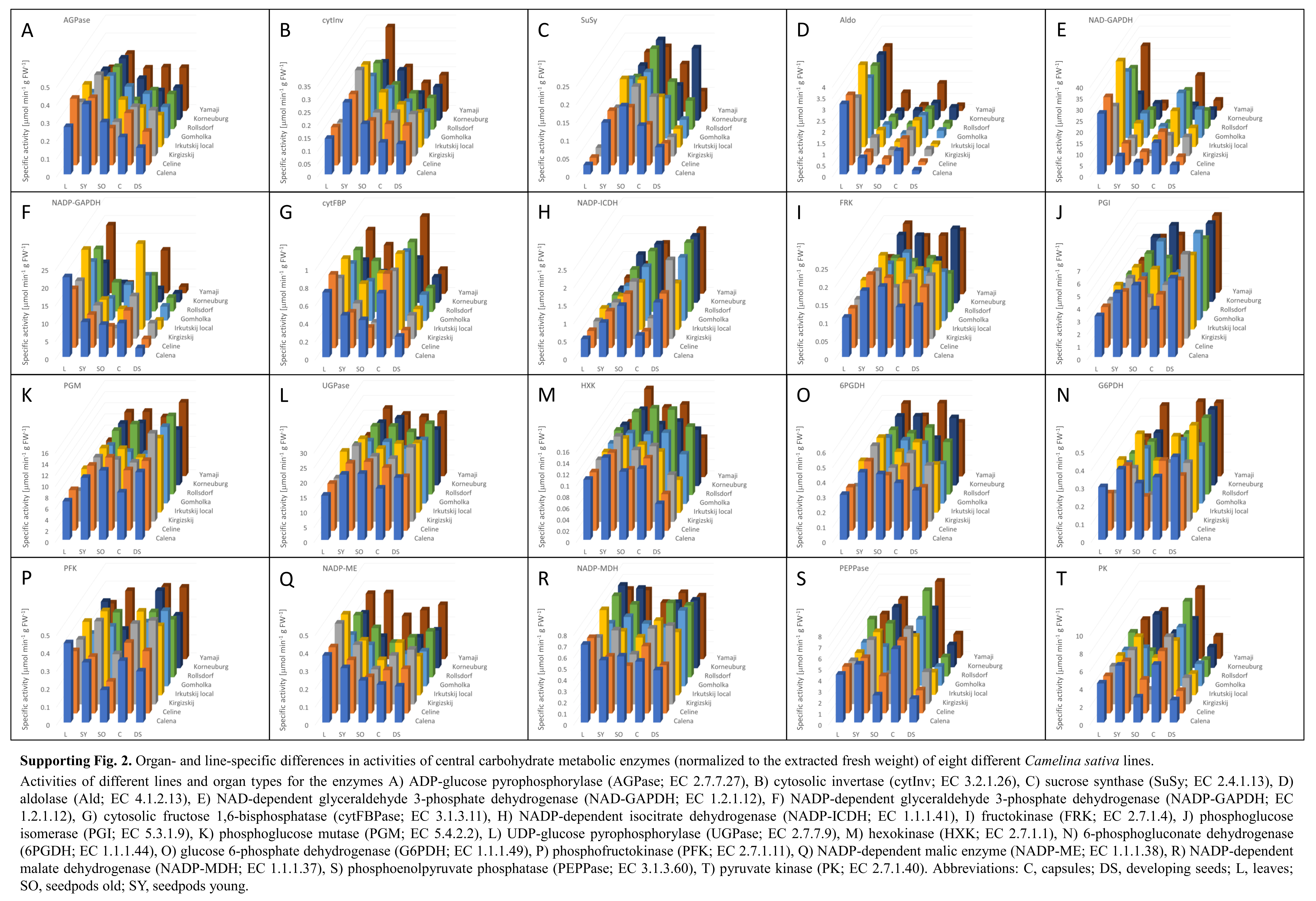

Supplement: Supplementary file 2 — Figure S2 [file FES3-12-e459-s009.tif]

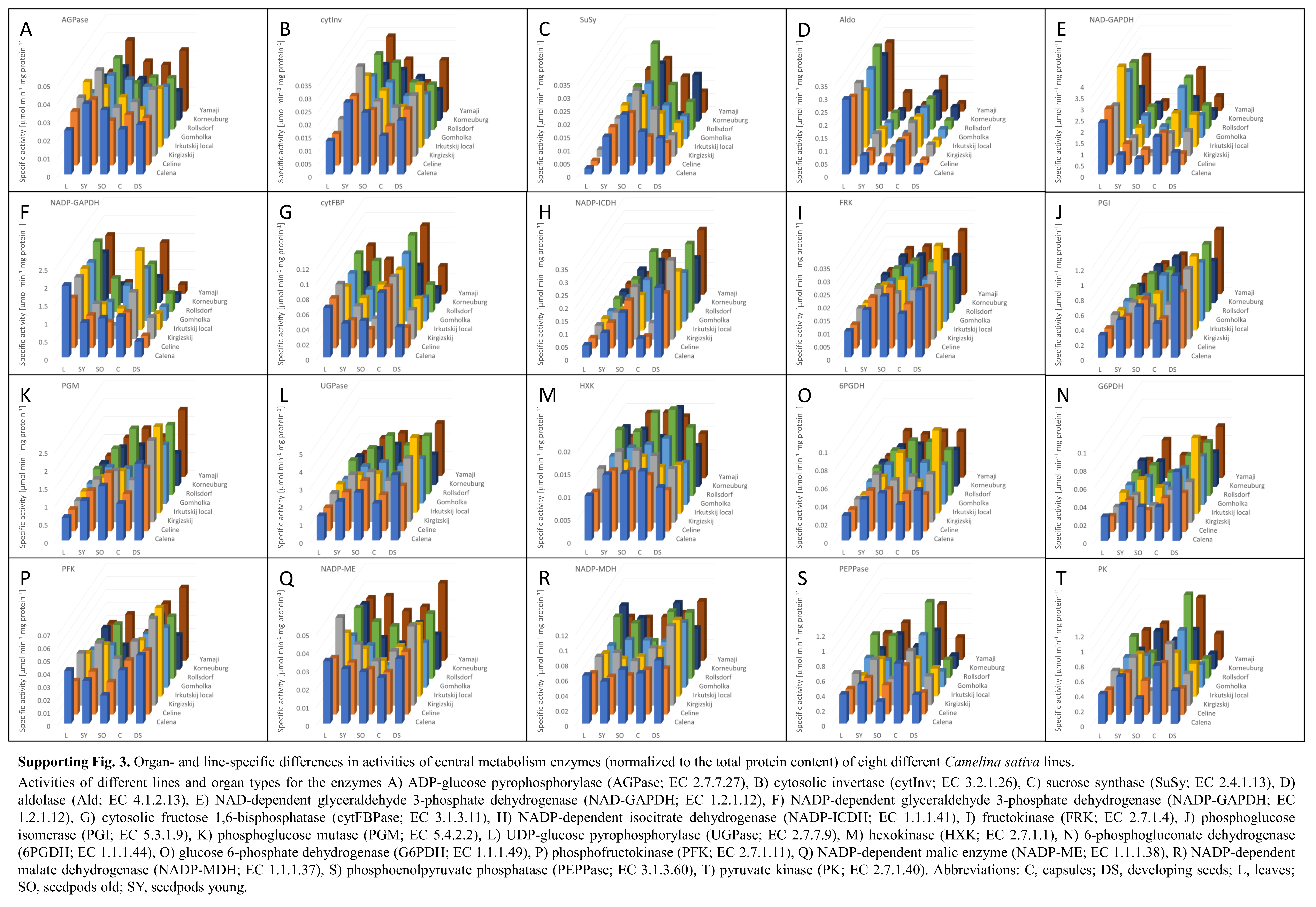

Supplement: Supplementary file 3 — Figure S3 [file FES3-12-e459-s010.tif]

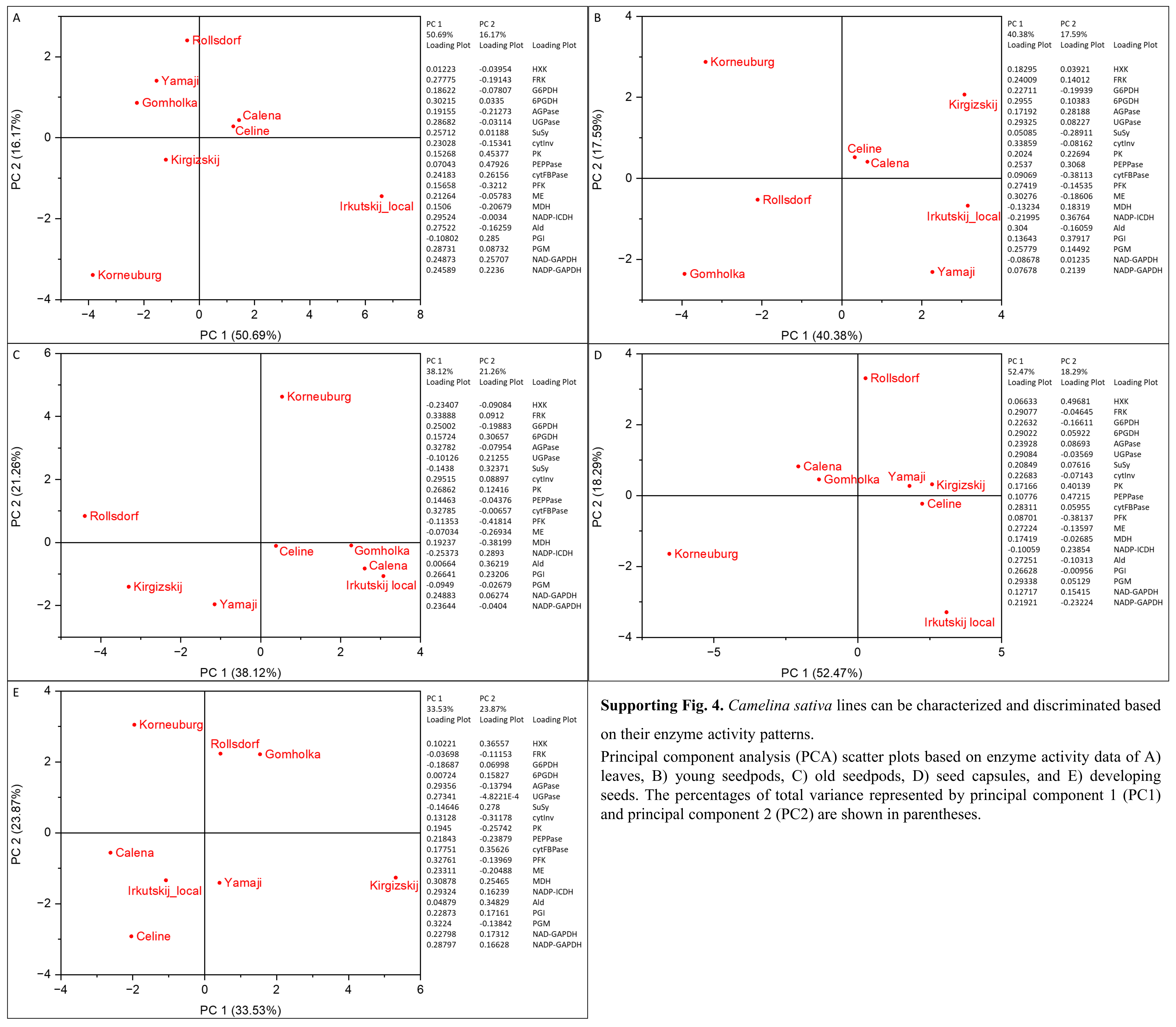

Supplement: Supplementary file 4 — Figure S4 [file FES3-12-e459-s005.tif]

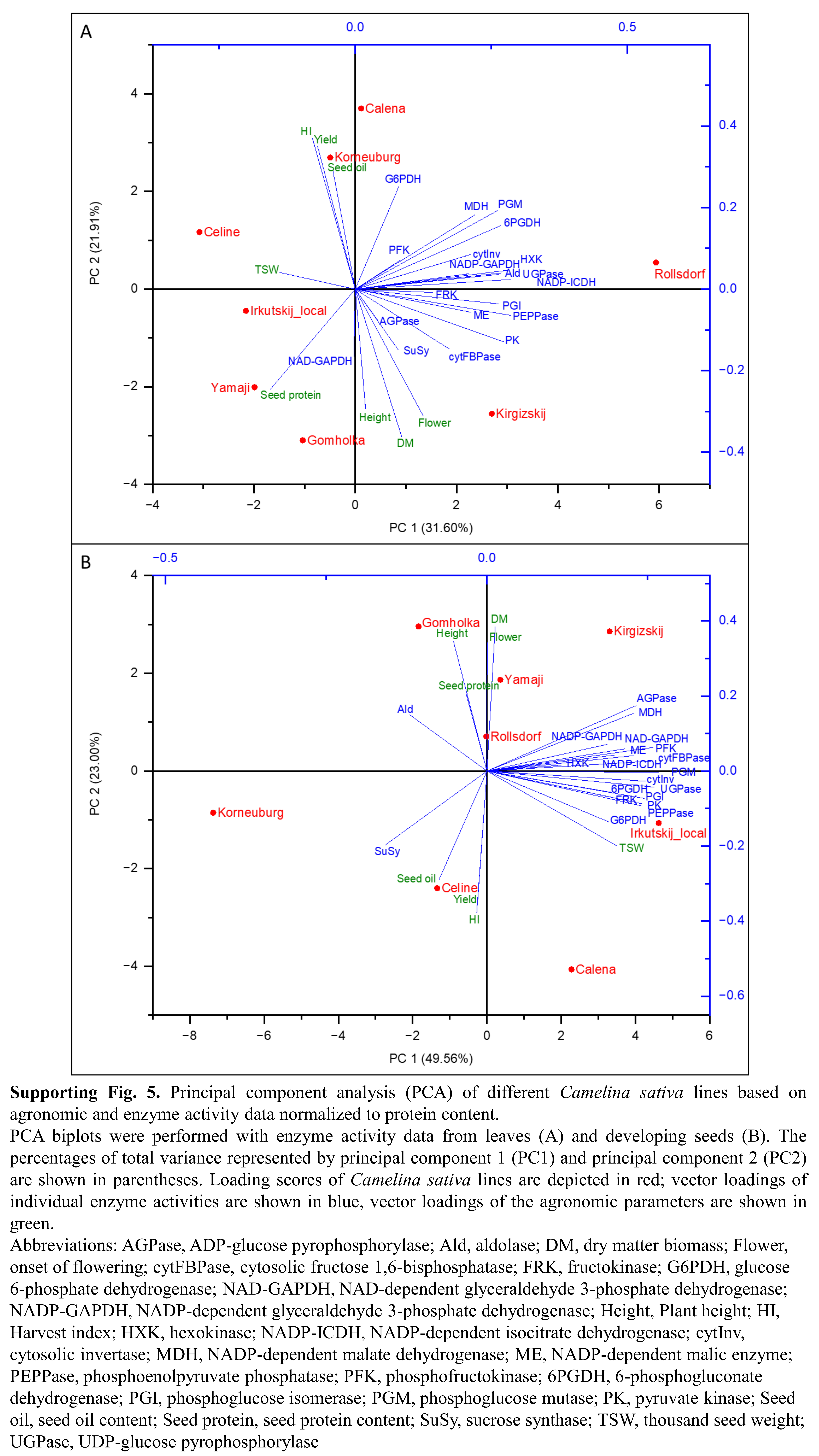

Supplement: Supplementary file 5 — Figure S5 [file FES3-12-e459-s011.tif]

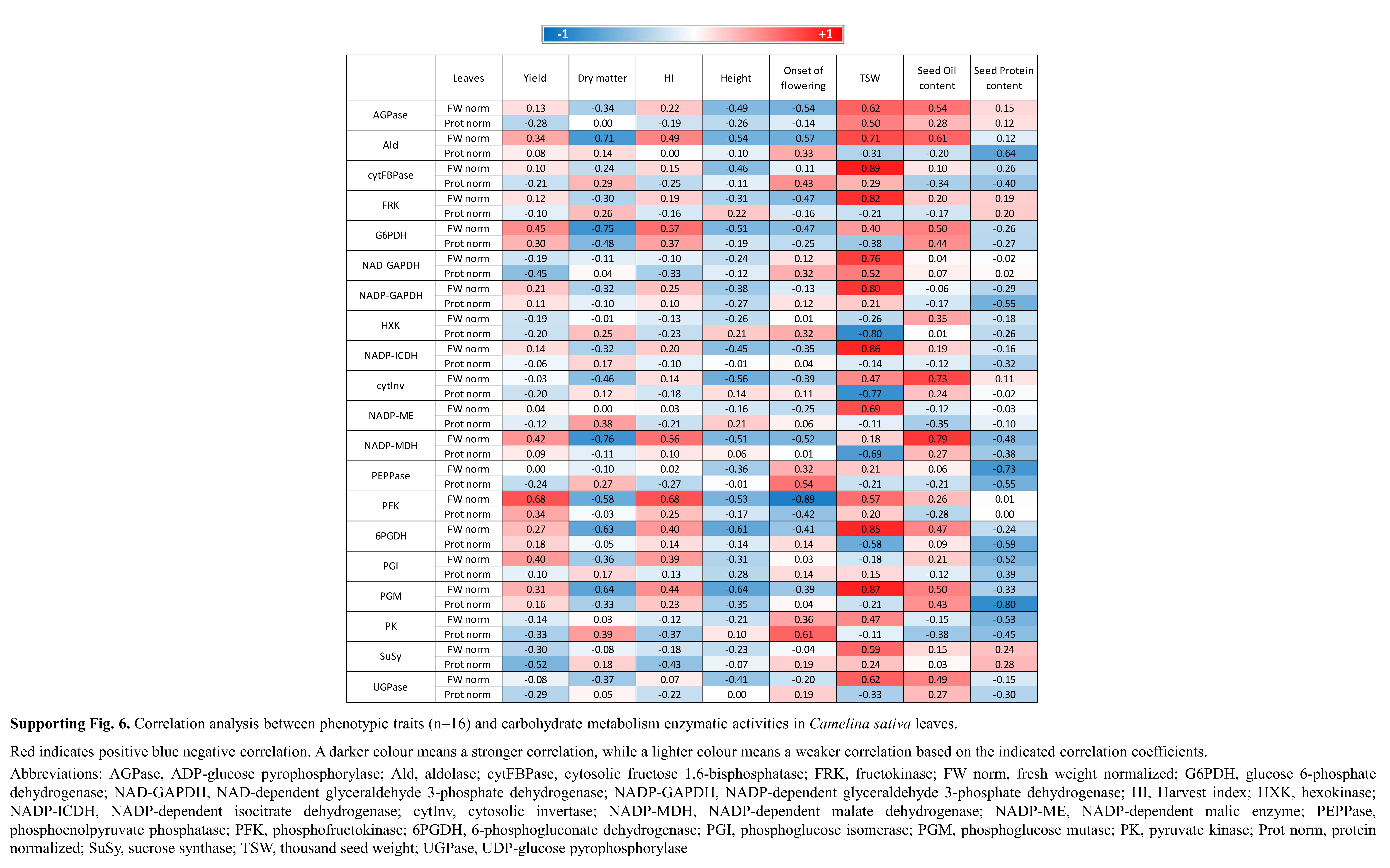

Supplement: Supplementary file 6 — Figure S6 [file FES3-12-e459-s008.tif]

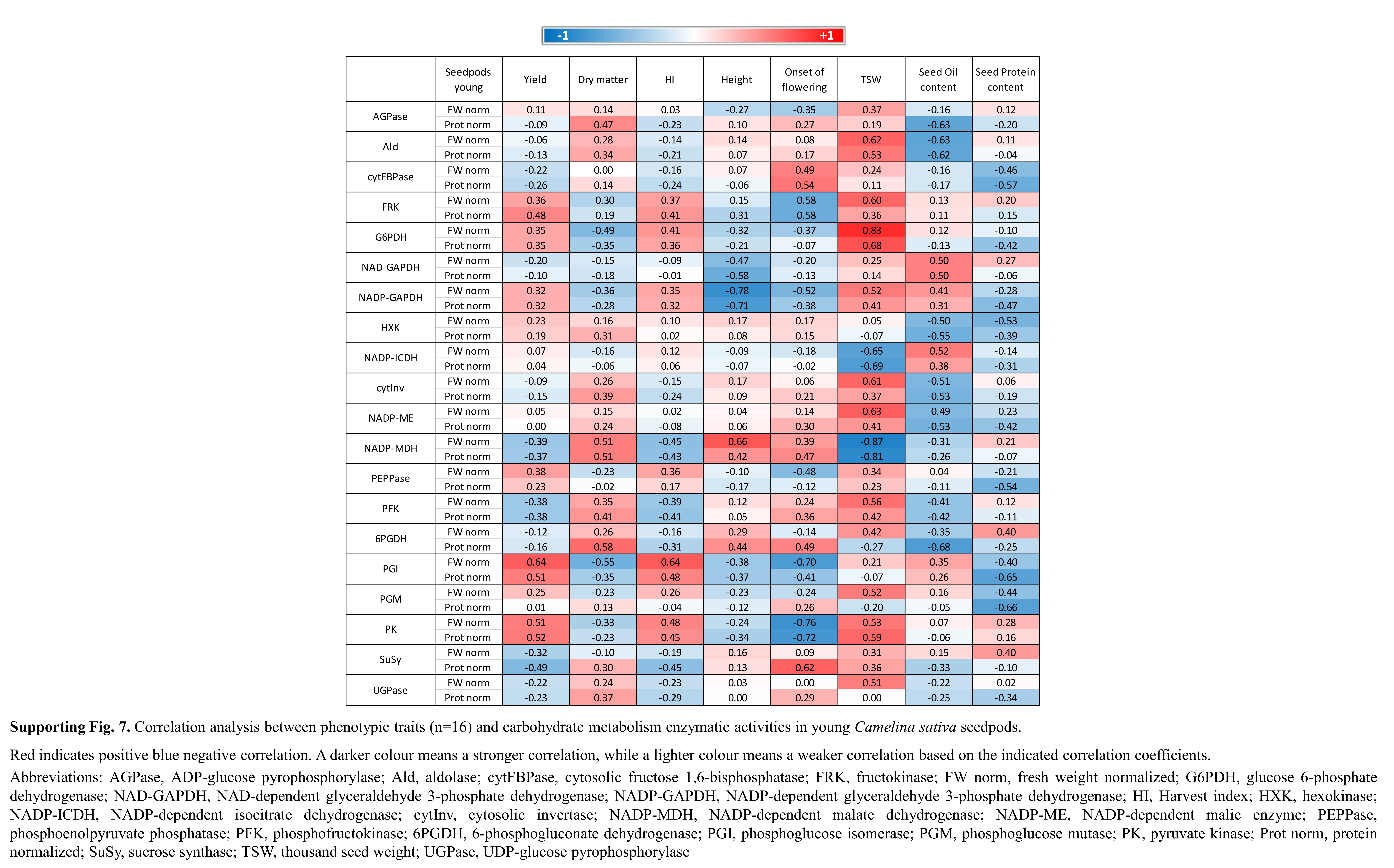

Supplement: Supplementary file 7 — Figure S7 [file FES3-12-e459-s004.tif]

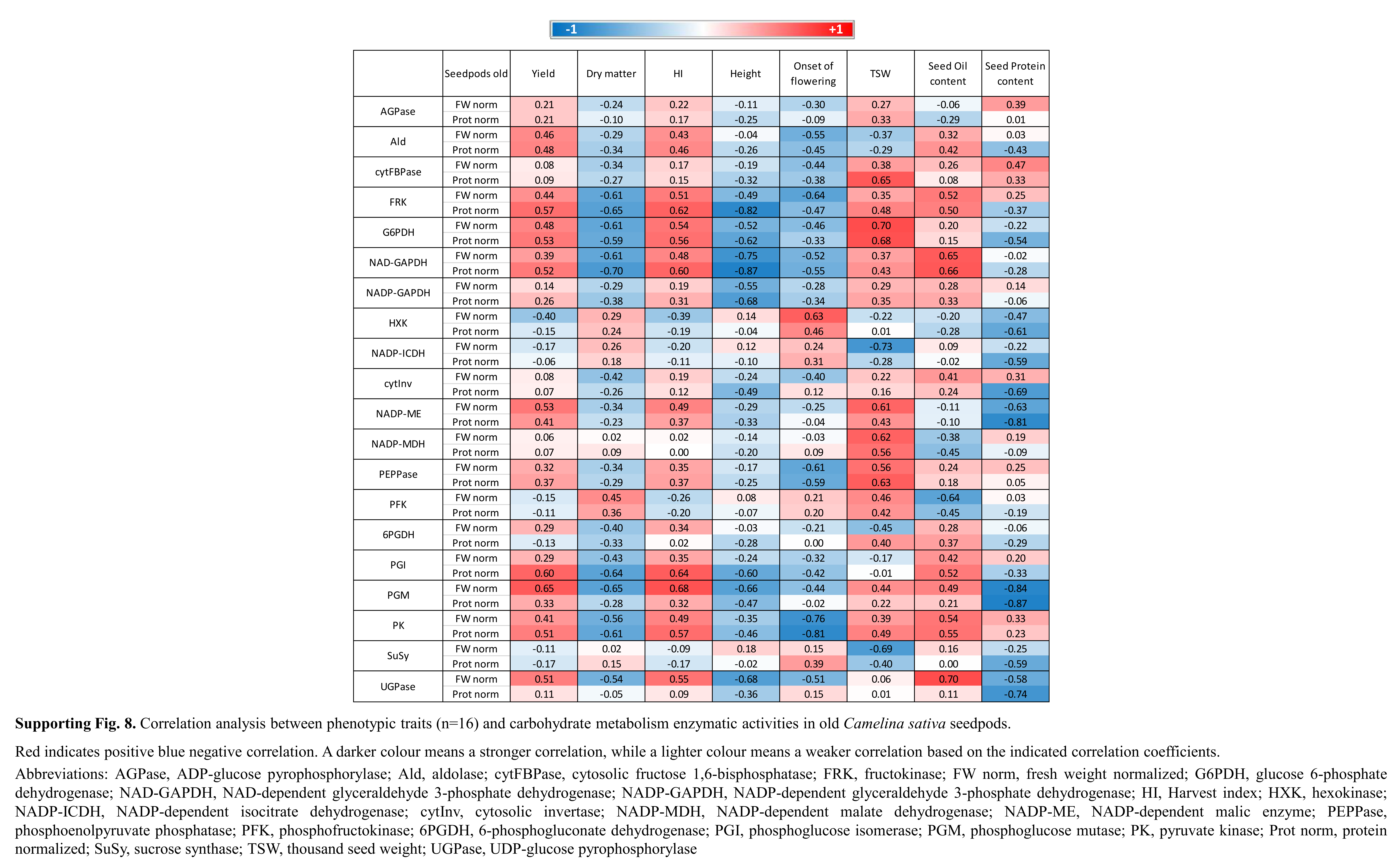

Supplement: Supplementary file 8 — Figure S8 [file FES3-12-e459-s006.tif]

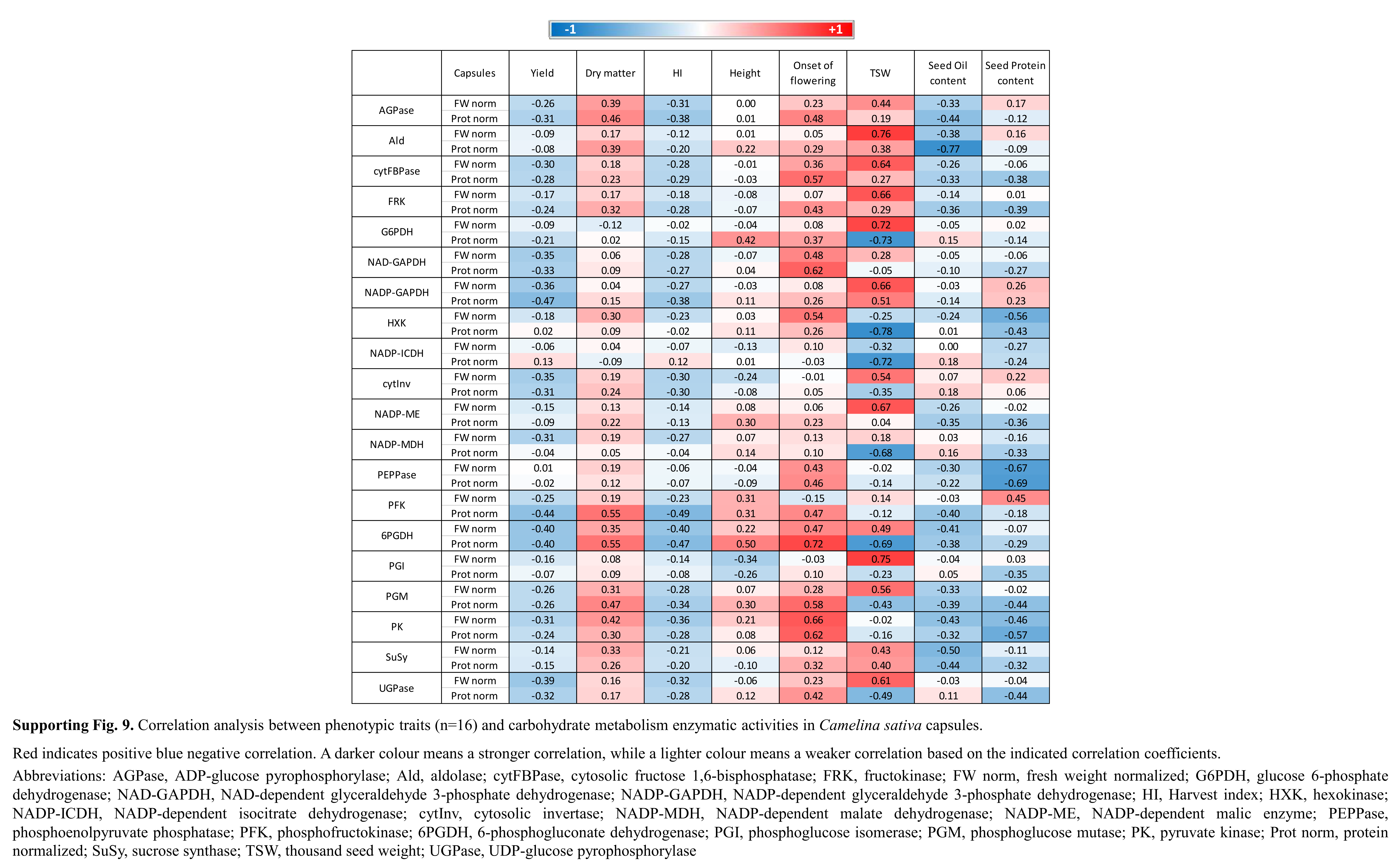

Supplement: Supplementary file 9 — Figure S9 [file FES3-12-e459-s002.tif]

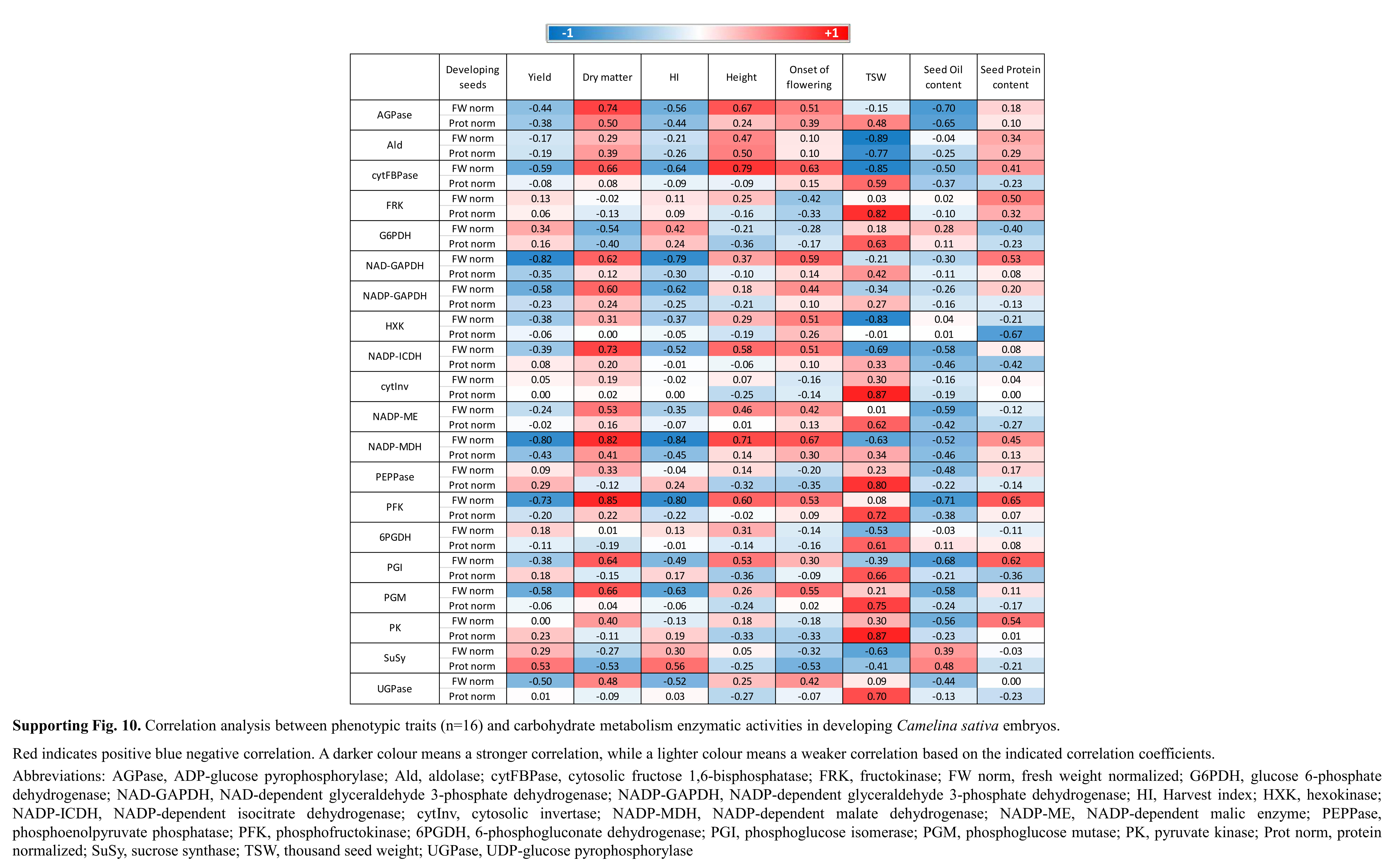

Supplement: Supplementary file 10 — Figure S10 [file FES3-12-e459-s007.tif]
